# DNMT1 regulates human erythropoiesis by modulating cell cycle and endoplasmic reticulum stress in a stage-specific manner

**DOI:** 10.1038/s41418-024-01305-6

**Published:** 2024-05-08

**Authors:** Qianqian Yang, Lixiang Chen, Hengchao Zhang, Mengjia Li, Lei Sun, Xiuyun Wu, Huizhi Zhao, Xiaoli Qu, Xiuli An, Ting Wang

**Affiliations:** 1 School of Life Sciences, Zhengzhou University, Science Road 100, Zhengzhou, 450001 China; 2 Department of Hematology, First Affiliated Hospital of Zhengzhou University, No.1 Jianshe East Road, Zhengzhou, 450052 China; 3 Laboratory of Membrane Biology, New York Blood Center, 310 East, 67th Street, New York, NY 10065 USA

**Keywords:** Epigenetics, Epigenetics

## Abstract

The dynamic balance of DNA methylation and demethylation is required for erythropoiesis. Our previous transcriptomic analyses revealed that DNA methyltransferase 1 (DNMT1) is abundantly expressed in erythroid cells at all developmental stages. However, the role and molecular mechanisms of DNMT1 in human erythropoiesis remain unknown. Here we found that DNMT1 deficiency led to cell cycle arrest of erythroid progenitors which was partially rescued by treatment with a p21 inhibitor UC2288. Mechanically, this is due to decreased DNA methylation of *p21* promoter, leading to upregulation of p21 expression. In contrast, DNMT1 deficiency led to increased apoptosis during terminal stage by inducing endoplasmic reticulum (ER) stress in a p21 independent manner. ER stress was attributed to the upregulation of RPL15 expression due to the decreased DNA methylation at *RPL15* promoter. The upregulated RPL15 expression subsequently caused a significant upregulation of core ribosomal proteins (RPs) and thus ultimately activated all branches of unfolded protein response (UPR) leading to the excessive ER stress, suggesting a role of DNMT1 in maintaining protein homeostasis during terminal erythroid differentiation. Furthermore, the increased apoptosis was significantly rescued by the treatment of ER stress inhibitor TUDCA. Our findings demonstrate the stage-specific role of DNMT1 in regulating human erythropoiesis and provide new insights into regulation of human erythropoiesis.

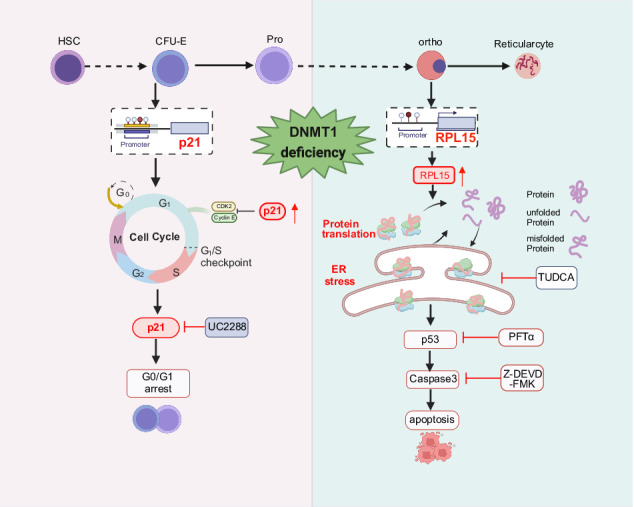

## Introduction

Erythropoiesis is the process by which hematopoietic stem cells (HSCs) proliferate and differentiate to produce mature red blood cells. The process consists of early erythropoiesis, terminal erythroid differentiation, and reticulocyte maturation. During early erythropoiesis, multipotent HSCs proliferate, differentiate, and generate erythroid progenitors, including burst-forming unit-erythroid (BFU-E) cells and colony-forming unit-erythroid (CFU-E) cells. During terminal erythroid differentiation, morphologically recognizable proerythroblasts undergo mitoses to become sequentially basophilic, polychromatic, and orthochromatic erythroblasts that then enucleate to become reticulocytes [1–4]. Proerythroblasts can divide 4 times, orthochromatic erythroblasts do not divide at all, which is accompanied by cell cycle exit [5, 6].

Erythropoiesis is a complex process that is tightly regulated by cytokines, transcription factors and other chromatin regulators [7–10]. DNA methylation is among the best-studied epigenetic modification, which provides a potential mechanism for maintaining cellular memory during cell divisions [11]. DNA methyltransferases (DNMTs) are responsible for establishing and maintaining genomic DNA methylation. DNMT1 copies the pattern of CpG methylation from the parental DNA strand to the daughter strand during the S phase, thereby maintaining the DNA methylation patterns. DNMT3A and DNMT3B are de novo methyltransferases [12]. Although global DNA demethylation occurs during both murine and human erythropoiesis and DNA demethylases (TETs) have been characterized to be essential for human erythropoiesis [13–15], maintaining methylation homeostasis is also critical for erythroid development [16–18]. Widespread changes in stage-specific patterns of DNA methylation occur, marking dynamic regulatory elements associated with patterns of gene expression that affect erythroid cell development and differentiation [17–20].

Our previous transcriptomic analyses of erythroid cells showed that DNMT1, but not DNMT3A or DNMT3B, was abundantly expressed in human erythroid cells. However, only the role DNMT1 in regulating γ-globin expression has been reported [21, 22]. The functional and mechanistic roles of DNMT1 in regulating erythropoiesis are not fully understood.

In the present study, we used a short hairpin RNA (shRNA)-mediated knockdown and a chemical DNMT1-specific inhibitor to study the effects and mechanisms of DNMT1 in the regulation of human erythropoiesis. We showed that DNMT1 deficiency led to cell cycle arrest by up-regulating p21 expression during early stage, and caused cell apoptosis by inducing ER stress during terminal erythroid differentiation. Our findings uncover the stage specific role of DNMT1 in human erythropoiesis and its underlying molecular mechanisms.

## Materials and methods

### Antibodies and reagents

The details of antibodies used in the present study are described in the supplementary file.

### Erythroid differentiation of CD34^+^ cells and small hairpin RNA-mediated knockdown

Primary human CD34^+^ cells from cord blood were isolated by positive selection using a CD34^+^ magnetic selective bead system (cat. no. 130-046-702, Miltenyi Biotechnology) as described in our previous studies [3, 4]. The purity of isolated CD34^+^ cells was 95–98%. The CD34^+^ cells were differentiated towards the erythroid lineage using a three-phase culture system. The culture medium composition, culture protocol, preparation of the lentivirus, and transduction in CD34^+^ cells have been described previously [14, 23].

### Drug treatments

The drugs for cell treatment were as follows. DNMT1 inhibitor DC_517 (cat. no. HY-12747, MCE) and endoplasmic reticulum (ER) stress inhibitor tauroursodeoxycholate TUDCA (cat. no. HY-19696A, MCE) dissolved in dimethyl sulfoxide (DMSO) were added into cell culture at a final concentration of 1 μM and 100 μM, respectively. Caspase-3 inhibitor Z-DEVD-FMK (cat. no. S7312, Sellleck) and p53 inhibitor pifithrin-α PFTα (cat. no. S2929, Selleck) was added into cell culture at a final concentration of 10 μM and 2 μM. p21 inhibitor UC2288 (cat. no. 532812, Millipore) was used at a final concentration of 1 μM.

### Flow cytometry analysis and fluorescence-activated cell sorting of erythroblasts

Early erythroid differentiation was monitored by analyzing the surface expression of glycophorin A (GPA), CD36, CD34, IL-3R as previously described [4]. Terminal erythroid differentiation was monitored by analyzing the surface expression of glycophorin (GPA), band 3, α4-integrin [3]. Apoptosis was assessed by 7AAD and Annexin V staining [14, 23, 24]. Stained cells were analyzed on a BD LSRFortessa flow cytometer and data analysis were performed using FCS Express 6 or Flow Jo software package. Erythroid cells at distinct differentiation stage were sorted using a MOFLO high-speed cell sorter (Beckman-Coulter) as previously described [15]. Sorted orthochromatic erythroblasts were subjected to RNA-seq, as described previously [3].

### Colony-forming assays

Cultured cells were plated in triplicate at a density of 200 cells in 1 ml of MethoCult H4434 classic medium (cat. no. 04434, STEMCELL) or in 1 mL of MethoCult H4330 medium with EPO only (cat. no. 04330, STEMCELL). The cells were incubated at 37 °C in a humidified atmosphere with 5% CO_2_. The BFU-E and CFU-E colonies were defined according to previously described criteria [4].

### Cytospin assay

Cells (0.1 × 10^6^) were suspended in 200 μL PBS and then adhered to a slide by Cytospin centrifugation. Slides were then stained with May-Grünwald (cat. no. MG500, Sigma) solution for 5 min. Then slides were washed with 40 mM tris buffer (pH 7.2) for 90 s, the slides were immediately stained with Giemsa solution (cat. no. GS500, Sigma) for 15 min. The images were taken using a standard light microscope (Axio Imager.A2, Carl Zeiss Microscopy GmbH, Jena).

### Global DNA methylation quantitation

Anti-5mC dot blot assays were performed as previously described [15]. Genomic DNA was extracted from 1 × 10^6^ cells using a Genomic DNA Mini Kit (Thermo Fisher Scientific). DNA was denatured for 5 min at 95 °C, placed on ice for 2 min, and two-fold serial dilutions were spotted on the activated polyvinylidene difluoride membrane with methyl alcohol. The blotted membrane was air-dried, UV-cross-link, blocked with 5% nonfat milk, and incubated with rabbit polyclonal anti-5mC (1:300) antibodies, as well as horseradish peroxidase–conjugated anti-rabbit immunoglobulin G secondary antibody (1:3000). Signal density was quantified using an Azure c600 imaging system (Azure Biosystems).

### O-Propargyl-puromycin flow analysis

Cells were plated at a density of 100,000 cells/ml and treated with 50 μM O-propargyl-puromycin (OP-Puro) (cat. no. HY-15680, MCE) for 1 h. Cells were washed twice, collected, and then subjected to processing using the Click-iT Flow Cytometry Assay kit (cat. no. C10418, Invitrogen) following the manufacturer’s instructions. Labeled cells were analyzed using a BD Fortessa instrument.

### RNA-seq and bioinformatics analyses

RNA was extracted from FACS-sorted orthochromatic erythroblasts following luciferase control and DNMT1 knockdown. Cells (1 × 10^6^) were collected and washed twice with PBS. RNA was extracted and used as the cDNA input, the cDNA library was prepared using a Truseq DNA library kit (Illumina), and the library was sequenced on a sequencing platform (HiSeq X Ten, Illumina). Reads with quality less than 30 and length less than 20 bp were trimmed using Trim Galore version 0.6.6. Trimmed reads were then mapped to the hg19 genome using TopHat version 2.0.13 (http://ccb.jhu.edu/software/tophat/) [25]. Gene expression was analyzed using the Cufflinks version 2.1.1 (http://cole-trapnell-lab.github.io/cufflinks/) package [26]. Fragments Per Kilobase of exon model per Million mapped fragments (FPKM) method was used for normalization. Principal component analysis (PCA) and correlation analysis were performed on expressed genes using the R software version 3.6.3 (https://www.r-project.org/) package. Differentially expressed genes (DEGs) were defined by FPKM > 10, *q* < 0.05, and fold change >2 using Cufflinks computational cuffdiff function. Gene Ontology (GO) enrichment analysis and visualization of DEGs was performed using the Metascape (www.metascape.org/) online tool with *q* < 0.05.

### Sodium bisulfite conversion and Bisulfite sequencing

Genomic DNA from cultured cells was isolated using TIANamp Genomic DNA Kit (cat. no. DP304-02, TIANGEN). DNA was quantified using Nanodrop (Thermo Fisher Scientific, MA, USA). DNA bisulfite conversion was performed using DNA Bisulfite Conversion Kit (cat. no. DP215-02, TIANGEN) according to manufacturer’s protocol. Briefly, 1 μg of DNA was mixed with 90 μL of Bisulfite Mix buffer in a final volume of 120 μL. Bisulfite conversion reaction was performed in a PCR instrument. Bisulfite-converted DNA was then column-purified using an adsorbent column and eluted in water. Bisulfite-converted DNA was quantified using Nanodrop. CpG island of target gene promoter (1000 bp upstream to 1000 bp downstream of transcription start site) and bisulfite sequencing primers were identified using MethPrimer (http://urogene.org/methprimer2/), the primer sequences are listed in Supplementary Table 2. PCR products were purified and cloned in T vector system (cat. no. 3271, Takara), which was then used to transform into the Tstbl3 Chemically Competent Cell (cat. no. TSC-C06, TSINGKE). The transformed colonies were then grown in LB medium with ampicillin. The plasmid was isolated using PurePlasmid Mini Kit (cat. no. CW0500M, CWBIO). Sequences were analyzed using the web-based tool QUantification tool for Methylation Analysis (QUMA, http://quma.cdb.riken.jp/).

### Statistical analyses

The experiments were performed 3 times independently and the repeated experiments showed similar results. Statistical analyses were performed with a two-tailed unpaired Student t test by Graphpad prism 8.0. Data were presented as mean ± standard deviation (S.D.). A difference was considered statistically significant at a value. *p < 0.05, **p < 0.01, ***p < 0.001.

## Results

### Expression of DNMTs during human erythroid differentiation

**Fig. 1:**
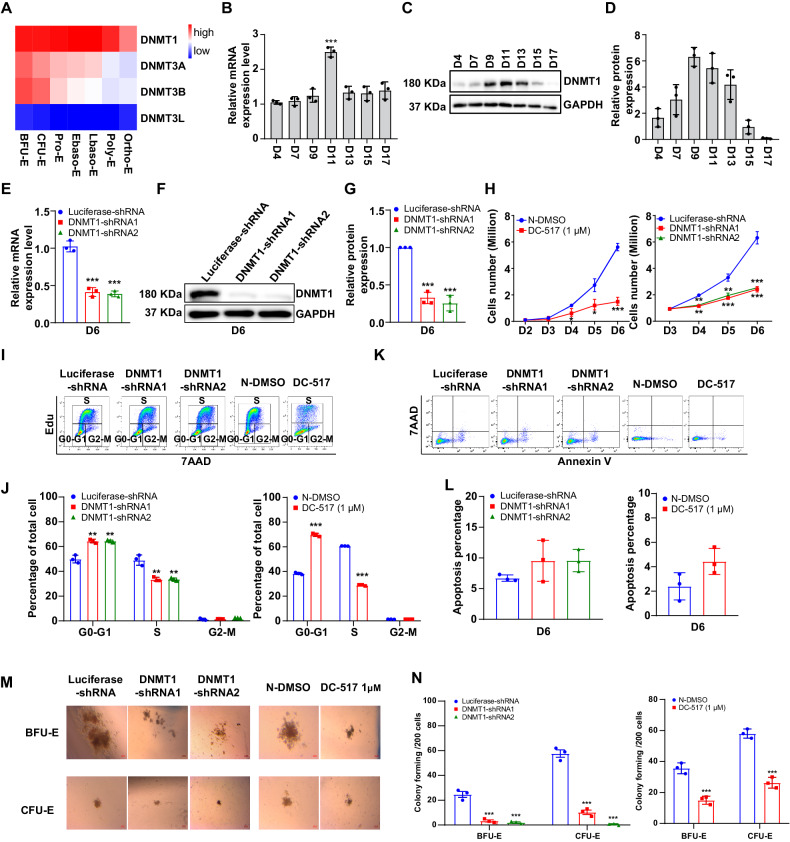
Effects of DNMT1 on proliferation of erythroid progenitors. **A** Heatmap showing mRNA levels of DNMTs as assessed by RNA-seq in erythroblasts. **B** qRT-PCR results showing DNMT1 mRNA expression levels in erythroblasts cultured for 4, 7, 9,11,13,15, and 17 days. GAPDH was used as internal reference. **C** Representative western blots showing DNMT1 protein levels in erythroblasts cultured for 4,7,9,11,13,15 and 17 days. **D** Quantitative analysis of DNMT1 protein levels from 3 independent experiments. **E** qRT-PCR results showing DNMT1 mRNA expression levels in luciferase-shRNA or DNMT1-shRNAs transduced erythroblasts cultured for 6 days. GAPDH was used as internal reference. **F** Representative western blot showing DNMT1 protein levels in luciferase-shRNA or DNMT1-shRNAs transduced erythroblasts cultured for 6 days. **G** Quantitative analysis of DNMT1 protein levels from 3 independent experiments. **H** Growth curves of Luciferase and DNMT1-knockdown and DNMT1 inhibitor erythroblasts at first 6 days. **I** Representative flow cytometry profiles of cell cycle as assessed by Edu and 7AAD staining of cells cultured for 6 days. **J** Quantitative analysis of cell cycle. **K** Representative flow cytometry profiles of apoptosis as assessed by dual staining of Annexin V and 7AAD of cells cultured for day 6. **L** Quantitative analysis of apoptosis from 3 independent experiments. **M** Colony-forming ability of cells cultured for day 6, which contain mixed populations of cells that include BFU-E, CFU-E, and proerythroblasts. **N** Colony number of Control (luciferase-shRNA and N-DMSO) and DNMT1-deficient (DNMT1-shRNAs and DC_517 1 µM) on day 6 of culture. * *p* < 0.05, ** *p* < 0.01, *** *p* < 0.001.

To evaluate the role of DNMTs during human erythropoiesis, we first analyzed the expression levels of DNMTs from the transcriptomics data of highly purified populations of erythroid cells from cultured cord blood at distinct stages of erythropoiesis [27]. Our transcriptomic analysis of erythroid cells revealed that DNMT3L were barely expressed through the whole process, DNMT3A and DNMT3B were expressed at a relatively high levels in early erythroblasts but downregulated during terminal stage. Notably, DNMT1 was the only member that was abundantly expressed at all differentiation stages (Fig. 1A). The expression of DNMT1 was further confirmed by real-time PCR (Fig. 1B) and western blot analysis (Fig. 1C, D). The constant and abundant expression of DNMT1 suggest a potentially important role for DNMT1 during human erythropoiesis.

### DNMT1 deficiency leads to reduced cell growth accompanied by cell cycle arrest in the G0/G1 phase in erythroid progenitors

To test our hypothesis, we used two methods to achieve DNMT1 dysfunction during early and terminal erythroid development. One strategy used shRNA/Cas9/dCas9-mediated approach to deplete the expression of DNMT1, and the second approach was to treat cells with DC_517, a specific inhibitor of DNMT1. First, we performed shRNA-mediated knockdown in human primary CD34^+^ cells in an in vitro erythroid cell culture system. Knockdown efficiency was examined on day 6 by quantitative reverse transcription-PCR analysis. Around 60% knockdown efficiency were achieved with both DNMT1-shRNA compared with control luciferase-shRNA (Fig. 1E). Western blot analysis revealed a ~70% reduction of DNMT1 at protein level (Fig. 1F, G). DNMT1 knockdown severely inhibited erythroid cell growth. Consistent with the phenotype of DNMT1-knockdown cells, the cell growth was significantly inhibited by DC_517 during the early stage (Fig. 1H). To investigate the reason for the impaired cell growth, we performed flow cytometry to examine the cell cycle using a 5-ethynyl-2’-deoxyuridine (Edu) incorporation assay. The representative profiles for the cell cycle analysis were shown in Fig. 1I. Quantitative analysis from 3 independent experiments showed that the G0/G1 population of both DNMT1-deficient cells and DC_517-treated cells were increased compared to control cells which was accompanied by decrease in S phase cells (Fig. 1J). These results indicate that the impaired cell growth of DNMT1-deficient early-stage cells is associated with cell cycle arrest at the G0/G1 phase.

In addition, we also performed CRISPR/Cas9 and CRISPR/dCas9 mediated genetic inactivation to further clarify and validate the role and mechanism of DNMT1 at the early stage. CRISPR-dCas9 mediated knockdown was performed to repress the expression of DNMT1 in human primary CD34^+^ cells and then induced to differentiate to erythroid cells. Real-time PCR revealed a ~90% knockdown efficiency of DNMT1 at day 6 (Supplementary Fig. 1A). Flow cytometry analysis showed that, compared with the control group, the dCas9 knockdown group showed an increase of more than 20% in G0/G1 phase and a decrease of more than 20% in S phase (Supplementary Fig. 1B, C). Furthermore, we also performed CRISPR/Cas9 mediated knockout of DNMT1 in HUDEP-2 cells. HUDEP-2 cells cultured under expansion growing (non-differentiating) was identified as erythroid progenitor cells. Western blot analysis revealed a ~90% reduction of DNMT1 expression at protein level (Supplementary Fig. 2A, B). Consistent with the phenotype of shRNA mediated DNMT1 knockdown cells, the cell growth was significantly inhibited by DNMT1 knockout during the early stage (Supplementary Fig. 2C). Flow cytometry analysis also revealed a more than 20% increase in G0/G1 phase population and a more than 20% decrease in S phase population in the DNMT1 knockout group (Supplementary Fig. 2D, E). Together, our findings demonstrate that DNMT1 deficiency severely impaired the cell expansion by arresting cell cycle at the G0/G1 phase.

Next, we checked apoptosis of the early-stage erythroid cells using Annexin V^+^ and 7AAD staining. The representative flow cytometry results were shown in Fig. 1K and Supplementary Fig. 1D. Quantitative analysis showed that there was no significant difference in the percentage of Annexin V^+^ cells between control and DNMT1 deficient or DC_517-treated primary erythroid progenitors (Fig. 1L, Supplementary Fig. 1E) and DNMT1 deficient HUDEP-2 cells (Supplementary Fig. 3A). The impaired proliferation of DNMT1 deficient erythroid progenitors was further demonstrated by the dramatic decreases in both the size and number of BFU-E and CFU-E colonies formed by DNMT1 deficient or DC_517 treated cells compared to their respective control cells (Fig. 1M, N). Supplementary Fig. 4A, B showed that DNMT1 knockdown or DC_517 treatment had no obvious effect on the differentiation of early-stage progenitors. Together these findings indicate that dysfunction of DNMT1 impairs cell growth by arresting cell cycle in G0/G1 phase without causing cell apoptosis.

### DNMT1 deficiency leads to decreased proliferation of erythroblasts

**Fig. 2:**
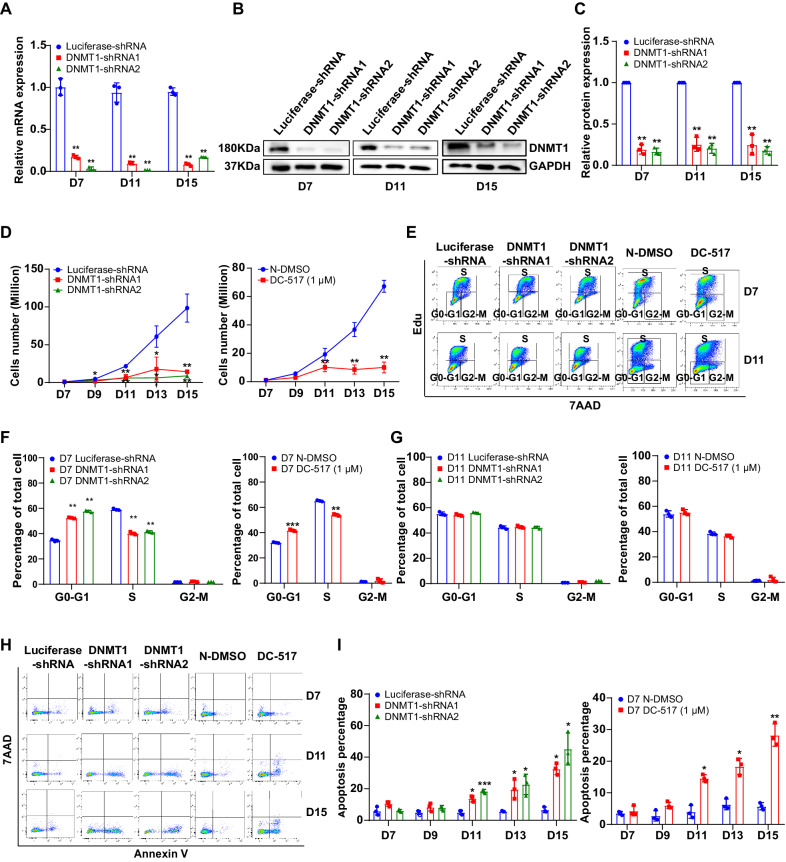
DNMT1 deficiency leads to cell number decrease of terminal erythroblasts. **A** qRT-PCR results showing DNMT1 mRNA expression levels in luciferase-shRNA or DNMT1-shRNAs transduced erythroblasts cultured for 7, 11, and 15 days. GAPDH was used as internal reference. **B** Representative western blot showing DNMT1 protein levels in luciferase-shRNA or DNMT1-shRNAs transduced erythroblasts cultured for 7, 11, and 15 days. **C** Quantitative analysis of DNMT1 protein levels from 3 independent experiments. **D** Growth curves of cells transduced with lentivirus containing luciferase-shRNA or DNMT1-shRNAs and N-DMSO or DNMT1 inhibitor cells for 7, 9, 11, 13, and 15 days. **E** Representative flow cytometry profiles of cell cycle as assessed by Edu and 7AAD staining of cells cultured for 7, 11 days. **F**, **G** Quantitative analysis of cell cycle. **H** Representative flow cytometry profiles of apoptosis as assessed by dual staining of Annexin V^+^ and 7AAD of cells cultured for 7, 9, 11, 13, and 15 days. **I** Quantitative analysis of apoptosis from 3 independent experiments. * *p* < 0.05, ** *p* < 0.01, *** *p* < 0.001.

We then explored the effects and mechanisms through which DNMT1 regulated terminal erythroid development. We first determined the knockdown efficiency of erythroblast at day 7, 11, and 15 using real-time PCR and western blot. A knockdown efficiency of greater than 50% for DNMT1-shRNA group was achieved (Fig. 2A–C). The differentiation of CFU-E cells to proerythroblasts is characterized by expression of the erythroid lineage-specific marker GPA. We thus checked the switch from early stage to terminal stage by determining GPA expression. We found that, on day 7, only ~15% of DNMT1 deficient cells were GPA-positive, while the proportion was ~40% in the control group (Supplementary Fig. 5A, B). Using the method we developed [2, 3], we further checked the differentiation of proerythroblasts to late-stage erythroblasts by monitoring the expression of surface markers α4-integrin and band 3. The representative flow cytometry results were shown in Supplementary Fig. 5C. Quantitative analysis showed that that DNMT1 deficiency slightly delayed terminal erythroid differentiation (Supplementary Fig. 5D). The delayed differentiation was confirmed by cytospin analyses which reveal that. DNMT1-deficient cells were larger than control cells (Supplementary Fig. 5E). Next, we checked the effect of DNMT1 deficiency on cell growth during terminal erythroid differentiation from day 7 to 15. Both knocking down DNMT1 and DC_517 treatment significantly impaired cell growth with the number of cells increasing from 1 × 10^6^ to ~10 × 10^6^, compared with the number of cells increasing from 1 × 10^6^ to ~80 × 10^6^ of the control group (Fig. 2D). To explore the mechanisms of the effect of DNMT1 deficiency on cell growth, we assessed the cell proliferation during terminal erythroid differentiation by monitoring cell cycle using an Edu incorporation assay from day 7 to 11. The representative profiles of the cell cycle analysis were shown in Fig. 2E. Quantitative analysis from 3 independent experiments showed that DNMT1 deficiency increased the G0/G1 stage population and decreased the S phase population on day 7 to 9 (Fig. 2F). However, the cell cycle did not change on day 11 (Fig. 2G). The results indicate that DNMT1 deficiency arrested the cell cycle before day 11. We also measured the extent of apoptosis. Representative profiles of dual staining with 7AAD and Annexin V^+^ of cells from day 7 to day 15 of culture were shown in Fig. 2H. Quantitative analysis showed that the Annexin V^+^ population was ~6% in luciferase-shRNA-transduced cells and ~45% in DNMT1-shRNA-transduced cells. The Annexin V^+^ population increased from 5% to 30% in DC_517-treated cells (Fig. 2I). We also analyzed the apoptosis of the dCas9 mediated DNMT1 knockdown primary erythroblast at days 7, 9, 11, 13, and 15. As shown in Supplementary Fig. 3B, the cell apoptosis rate was significantly increased in the dCas9-DNMT1 knockdown group. Next, we checked apoptosis of the DNMT1 deficient HUDEP-2 cell under differentiation conditions. As shown in Supplementary Fig. 3A, after inducing to differentiate for 4, 6, and 8 days, cell apoptosis of DNMT1 knock out HUDEP-2 cell was also significantly increased. Taken together, these findings demonstrate that different from the early stage, DNMT1 deficiency impaired proliferation of terminal erythroblasts via both cell cycle arrest and apoptosis.

### Cell cycle arrest of DNMT1-deficient erythroid progenitors is associated with decreased promoter methylation and subsequent upregulation of p21

**Fig. 3:**
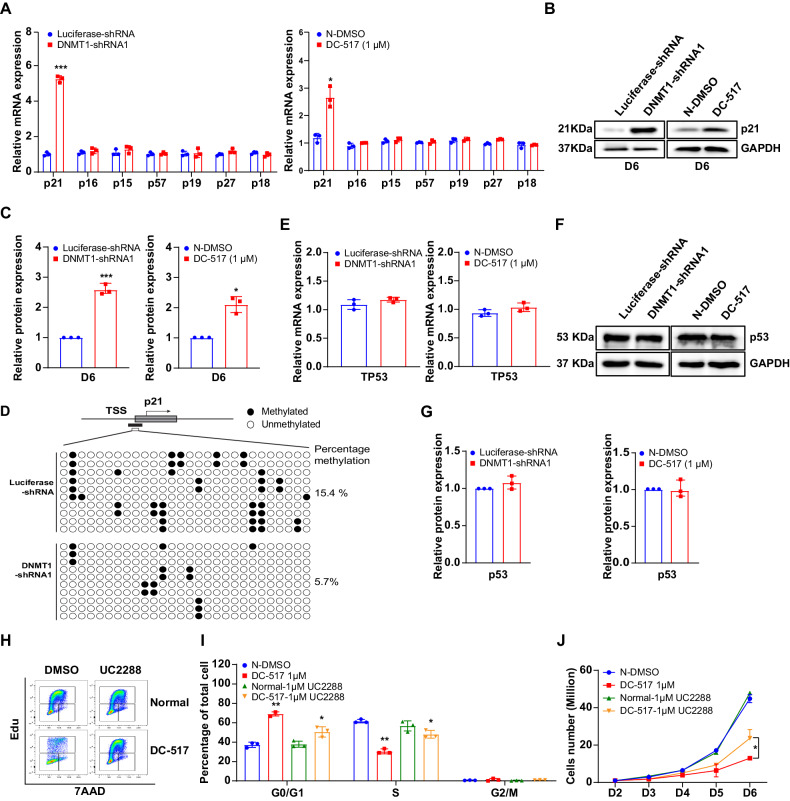
DNMT1 deficiency led to cell cycle arrest due to reduction in methylation level of p21. **A** mRNA levels of p21, p27, p57, p15, p16, p18, and p19, as assessed by qRT-PCR. GAPDH was used as internal reference. **B** Representative western blot analysis of p21. GAPDH was used as loading control. **C** Quantitative analysis of p21 from 3 independent experiments. **D** Bisulfite sequencing of cells. **E** mRNA levels of p53, as assessed by qRT-PCR. **F** Representative western blot analysis of p53. GAPDH was used as loading control. **G** Quantitative analysis of p53 from 3 independent experiments. **H** Representative flow cytometry profiles of cell cycle as assessed by Edu and 7AAD staining of N-DMSO and 1 μM DC_517 cells cultured for 6 days in the presence of DMSO or 1 μM UC2288. **I** Quantitative analysis of cell cycle. **J** Growth curves of N-DMSO and DC_517 1 μM cells in the presence of DMSO or 1 μM UC2288. * *p* < 0.05, ** *p* < 0.01, *** *p* < 0.001.

It has been well established that cyclins and cyclin-dependent kinases (CDKs) form cyclin-CDK complexes to drive cell-cycle progression [28], whereas cyclin-dependent kinase inhibitors (CDKIs) promote cell cycle arrest by inhibiting CDK activity [29]. CDKI family members include p21, p27, p57, p15, p18, and p19, which are essential in regulating the cell cycle [28–31]. We reasoned that DNMT1 deficiency-induced cell cycle arrest could be due to aberrant expression of CDKIs. To test this, we checked the mRNA levels of CDKI family members in control and DNMT1-deficient erythroid cells. As shown in Fig. 3A, the mRNA level of p21 increased significantly (~5 fold) in DNMT1-knockdown cells on day 6, compared with that of the control group. In addition, the mRNA level of p21 in DC_517-treated cells also increased 2 fold (Fig. 3A). The increased p21 was confirmed at the protein level by western blot result (Fig. 3B, C). DNA methyltransferase modulates gene expression by regulating DNA methylation status at the promoter region by catalyzing methylation at the 5′-position of cytosine. Next, we examined 5mC level by dot blot analysis. The 5mC level decreased in day 6 DNMT1-knockdown erythroid cells compared with luciferase-shRNA cells (Supplementary Fig. 6A, B). It was consistent with the changes we observed in the shRNA-mediated DNMT1 knock down erythroid progenitors, we found that Cas9-mediated knock out of DNMT1 also lead to a significant decrease of 5mC level in HUDEP2 cells cultured in expansion medium (Supplementary Fig. 6C, D). To examine whether increased p21 expression in DNMT1 deficient cells was associated with methylation changes of *p21* promoter, we examined the methylation status of *p21*. The CpG islands of promoter region of *p21* were predicated using MethPrimer (Supplementary Fig. 6E). By conducting Bisulfite sequencing, we found that there were 28 CpG sites of *p21* promoter region named pos1-pos28 (Supplementary Fig. 6F). Among the 28 CpG sites, 15 CpG sites were methylated in control cells while only 7 CpG sites were methylated in DNMT1-deficient cells (Fig. 3D). Compared with the methylation level of 15.4% in control cells, the methylation level decreased to 5.7% in DNMT1-deficient cells (Fig. 3D). By analyzing the methylation percentages of each CpG site, we found that DNMT1 deficiency caused dramatically decrease of the methylation of some specific CpG sites, such as site 3, 7, 13, 14, 18, 21, 22, 23, 25, 27, and 28, while showed no significant effect on the methylation of the other sites (Supplementary Fig. 6G). This result indicated that DNMT1 regulated the DNA methylation of *p21* in a CpG site-specific manner. Notably, the expression of *TP53* was not affected by DNMT1 deficiency (Fig. 3E–G). Our findings suggested that *p21* could be a direct target of DNMT1. To clarify whether p21 played a role in cell cycle arrest due to DNMT1 deficiency, we treated the DNMT1-deficient cells with the p21 inhibitor, UC2288, and checked the cell cycle. Figure 3H, I showed that UC2288 treatment decreased the G0/G1 cell population (10–20%) and increased the S population (~ 20%) of the DC_517-treated cells. Moreover, UC22888 treatment also partially restored the growth of DNMT1-deficient cells (Fig. 3J). Taken together, these results demonstrate that p21 acts as the key factor that mediates the roles of DNMT1 in the regulation of cell cycle during early stage of human erythropoiesis.

### Upregulation of core ribosomal proteins in DNMT1-deficient orthochromatic erythroblasts due to decreased promoter methylation and subsequent upregulation of RPL15

**Fig. 4:**
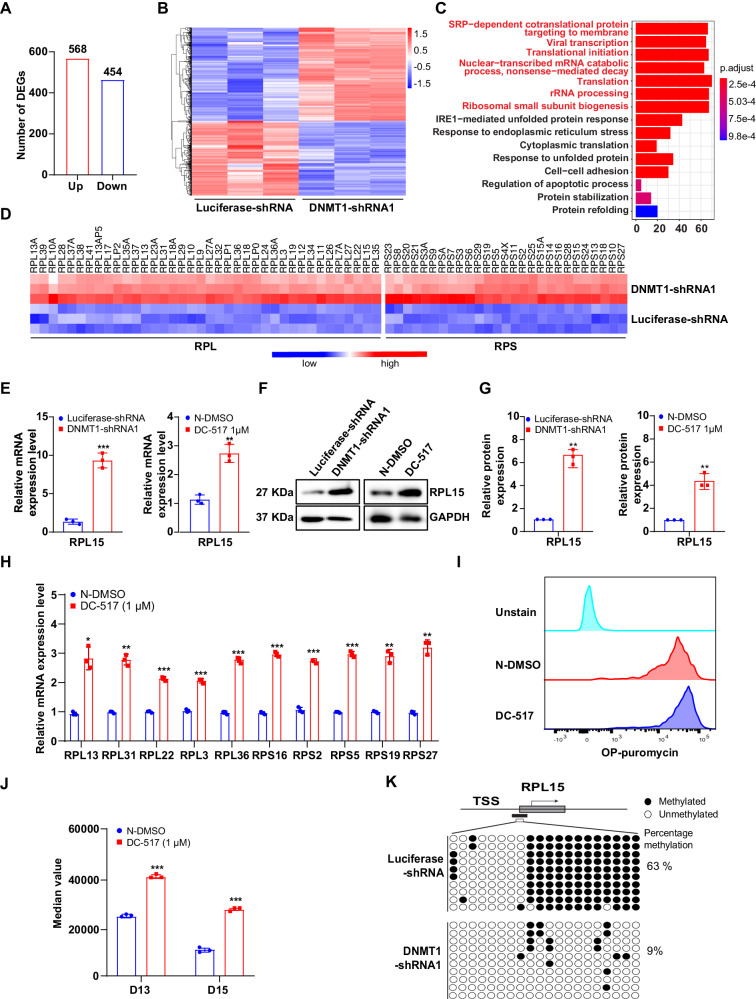
DNMT1 deficiency increases RPL15 expression by altering methylation levels in the *RPL15* promoter region. **A** Number of differentially expressed genes among luciferase-ortho, DNMT1-KD-ortho. as revealed by RNA-seq analysis. **B** Heatmap of the differentially expressed genes. **C** The top 15 upregulated pathways in luciferase ortho, DNMT1-knockdown ortho, and *P* value are shown. **D** The FPKM value of core RPs. **E** qRT-PCR results showing *RPL15* mRNA expression levels in DNMT1 deficiency erythroblasts cultured for 15 days. GAPDH was used as internal reference. **F** Representative western blot analysis of RPL15. GAPDH was used as loading control. **G** Quantitative analysis of RPL15 protein levels from 3 independent experiments. **H** qRT-PCR results showing RPs mRNA expression levels in DC_517 treated erythroblasts cultured for 15 days. GAPDH was used as internal reference. **I** Increased global translation in DNMT1 deficiency erythroblast. Representative histograms and quantification of OP-Puro incorporation into control and DC_517 treated cells. **J** Quantitative analysis from 3 independent experiments showing the median value of fluorescence. **K** Bisulfite sequencing analysis of CpG islands in the promoter region of *RPL15*. Methylated (black circle) and unmethylated (white circle) CpG dinucleotides are displayed. Shown here are the percentages of methylation in DNMT1-deficient cell and normal erythroid cells (63% and 9%, respectively). * *p* < 0.05, ** *p* < 0.01, *** *p* < 0.001.

Next, we thought to explore the molecular mechanisms for the DNMT1 deficiency-induced apoptosis. We found that DNMT1 knockdown resulted in greater reduction of the level of 5mC in shRNA-mediated DNMT1 knock down erythroblasts compared with control group (Supplementary Fig. 7A, B). It was consistent with the changes we observed in the shRNA-mediated DNMT1 knock down erythroblast, we found that Cas9-mediated knock out of DNMT1 also lead to a significant decrease of 5mC level in HUDEP-2 cells induced for 8 days (Supplementary Fig. 7C, D). The p21 inhibitor UC2288 partially rescued cell cycle arrest induced by DNMT1 deficiency in the early stage of erythropoiesis, but failed to rescue apoptosis induced by DNMT1 deficiency in the terminal stage (Supplementary Fig. 8A–D), which suggests that apoptosis induced by DNMT1 deficiency may not be reliant on p21. In addition, we also checked DNA methylation of *p21* promoter at the terminal stage. As shown in Supplementary Fig. 8E, there was no significant difference of the DNA methylation levels between the control group and DNMT1 deficient group, further confirming that DNMT1 regulating the cell expansion in a p21 independent manner during terminal stage. To determine the molecular mechanism for the DNMT1 deficiency-induced apoptosis, we performed RNA-seq analysis on the purified luciferase-shRNA and DNMT1-knockdown orthochromatic erythroblasts. Bioinformatics analysis revealed that DNMT1 knockdown resulted in upregulation of 568 genes and downregulation of 454 genes (Fig. 4A). Figure 4B showed the heatmap of differentially expressed genes. Gene Ontology analysis of the upregulated genes revealed a subset with strong ribosome and protein synthesis signatures (Fig. 4C). Specifically, a large number of core ribosomal proteins (RPs) were significantly increased (Fig. 4D). RPs are essential components of large and complex structures and their expression is highly coordinated to ensure accurate biogenesis and assembly of ribosome subunits. Previous studies have shown that overexpression of RPL15 leads to coordinated upregulation of core RPs [32, 33]. Therefore, we hypothesized that DNMT1 deficiency might alter the expression of RPs by regulating the expression of RPL15. Both real-time PCR (Fig. 4E) and western blot (Fig. 4F, G) analysis demonstrated that mRNA and protein levels of RPL15 were upregulated in DNMT-deficient orthochromatic erythroblasts. We also verified the upregulated mRNA levels of top 5 ribosomal proteins (RPLs and RPSs) using real-time PCR (Fig. 4H). Moreover, DNMT1-deficient erythroblasts exhibited increased global translational activity as assessed by O-propargyl-puromycin (OPP) labeling assay (Fig. 4I). To define the molecular mechanism for the DNMT1 deficiency-induced upregulation of RPL15, we used the MethPrimer online tool to predict CpG islands in the promoter region of *RPL15* and identified 3 CpG islands (Supplementary Fig. 7E). We further examined the methylation status of the *RPL15* promoter region using BSP, which revealed that among the 20 CG sites, 16 CG sites were methylated in luciferase-shRNA cells while only 8 CG sites were methylated in DNMT1-deficient cells (Fig. 4K). Furthermore, the methylation level was 63% in luciferase-transduced cells, but only 9% in DNMT1-shRNA-transduced cells (Fig. 4K). These results indicate that knockdown of DNMT1 leads to an overactivation of the translation process in erythroblasts via the upregulation of RPL15.

### Total ER stress-mediated pathways are increased following DNMT1 knockdown in late erythroblasts

**Fig. 5:**
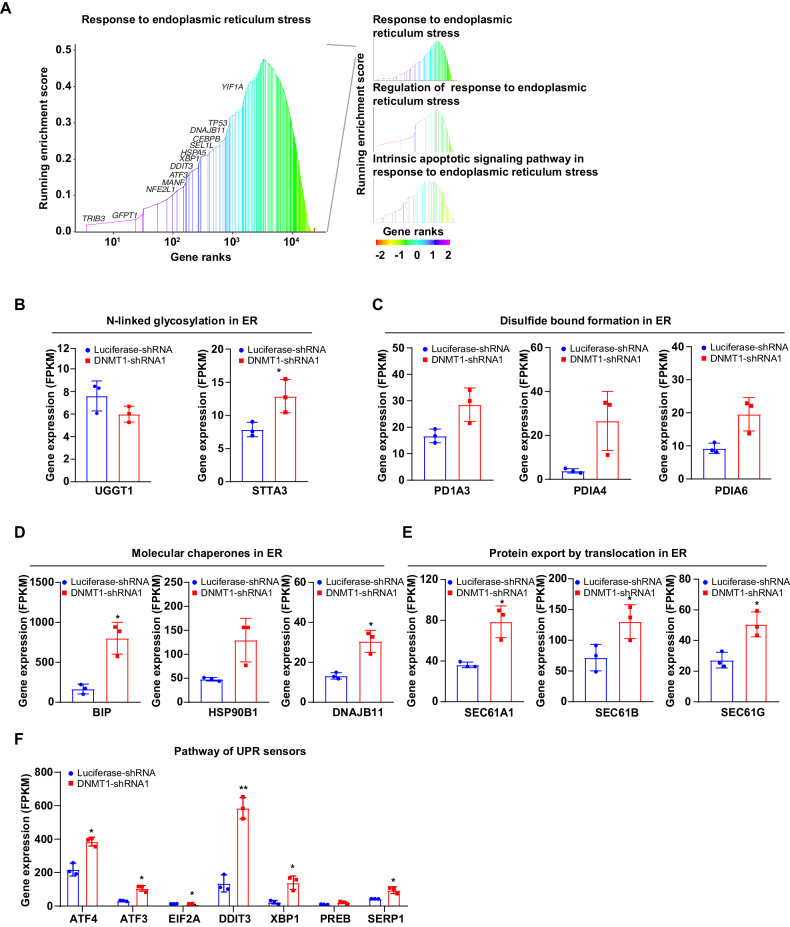
DNMT1 regulate ER stress related-gene in late stage of terminal erythroblasts. **A** Rank-based gene set enrichment pathways by DNMT1 significantly regulated. The images of GSEA demonstrated the key pathways which all involved in ER stress. **B** The FPKM value of N-linked glycosylation in ER. **C** The FPKM value of Disulfide bound formation in ER. **D** The FPKM value of Molecular chaperones in ER. **E** The FPKM value of protein export by translocation in ER. **F** The FPKM value of pathway of UPR sensor and downstream molecular in ER. * *p* < 0.05, ** *p* < 0.01.

Early works have revealed that upregulation of translation increases ribosome biogenesis and protein load in the endoplasmic reticulum (ER), thereby activating all branches of the UPR [34, 35]. Our Gene Ontology and GSEA analysis showed that, in the DNMT1-deficient orthochromatic erythroblasts, all ER stress pathways including IRE1-mediated unfolded protein response, ATF6-mediated unfolded protein response, PERK-mediated unfolded protein response were activated (Fig. 4C and Fig. 5A). Specifically, following genes were significantly increased in DNMT1-knockdown orthochromatic erythroblasts compared to control cells: UGGT1 and STT3A involved in N-linked glycosylation ER stress (Fig. 5B). PDIA3, PDIA4, and PDIA6 involved in N-linked glycosylation ER stress (Fig. 5C). BIP, HSP90B1, and DNAJB11, which were molecular chaperones involved in ER stress (Fig. 5D). SEC61AI, SEC61B, and SEC61G involved in protein export by translocation (Fig. 5E); ERN1 and EIF2A, which were unfolded protein response sensors (Fig. 5F), and ATF4, ATF3, DDIT3, XBP1, PREB, SERP1, and other genes for molecules involved in ER stress downstream pathways (Fig. 5F, Supplementary Fig. 9A). To test if the ER stress-mediated pathway was specially activated in response to the deficiency of DNMT1 at the terminal stage, we conducted qRT-PCR to evaluate mRNA levels of the ER stress-related genes BIP, DDIT3, ATF4, and XBP1 in both the control group and the DNMT1 deficient group during the early stage of erythropoiesis (Supplementary Fig. 9B and C). We found that DNMT1 deficiency does not affect the expression of ER stress-related genes during early stage of erythropoiesis, suggesting that DNMT1 deficiency lead to activation of ER stress especially at the terminal stage of erythropoiesis.

### DNMT1 deficiency activated-ER stress pathway leads to apoptosis in late stage of terminal erythroblasts

**Fig. 6:**
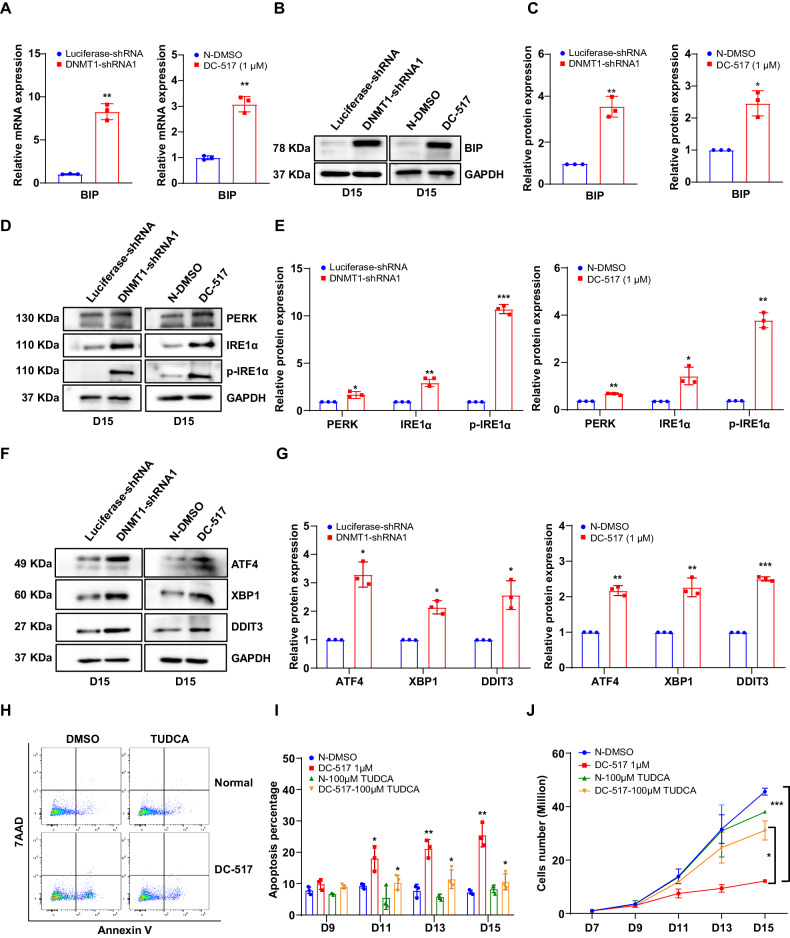
DNMT1 deficiency activates ER stress pathway in late stage of terminal erythroblasts. **A** qRT-PCR results showing BIP mRNA expression levels in DNMT1 deficiency erythroblasts cultured for 15 days. GAPDH was used as internal reference. **B** Representative western blot analysis of BIP. GAPDH was used as loading control. **C** Quantitative analysis of BIP protein levels from 3 independent experiments. **D** Representative western blot analysis of PERK, IRE1a, p-IRE1a. GAPDH was used as loading control. **E** Quantitative analysis of PERK, IRE1α, p-IRE1α from 3 independent experiments. **F** Representative western blot analysis of ATF4, XBP1, DDIT3. GAPDH was used as loading control. **G** Quantitative analysis of ATF4, XBP1, DDIT3 from 3 independent experiments. **H** Representative flow cytometry profiles of apoptosis as assessed by Annexin V and 7AAD staining of Normal-DMSO and 1 μM DC_517 cells cultured for 15 days in the presence of DMSO or 100 μM TUDCA. **I** Quantitative analysis of apoptosis from 3 independent experiments. **J** Growth curves of Normal-DMSO and 1 μM DC_517 cells in the presence of DMSO or 100 μM TUDCA for 7, 9, 11, 13 and 15 days. * *p* < 0.05, ** *p* < 0.01, *** *p* < 0.001.

ER stress sensor BIP has been widely reported to act as the primary sensor in the activation of the UPR response [36, 37]. We then checked the mRNA and protein levels of BIP in DNMT1-deficient orthochromatic erythroblast using real-time PCR and western blot (Fig. 6A–C) and found that the mRNA and protein levels of BIP were significantly upregulated following DNMT1 knockdown. To further confirm the excessive activation of ER stress, we examined the protein levels of ER sensor PERK, IRE1α and phosphorylation of IRE1α by western blot. As shown in Fig. 6D, E, the protein levels of PERK, IRE1α and the phosphorylation of IRE1α were all increased significantly in DNMT1 deficient orthochromatic erythroblasts. Moreover, we detected mRNA and protein levels of ER stress downstream molecules ATF4, X-box binding protein 1 (XBP1), and DNA damage-inducible transcript 3 (DDIT3) by Real-time PCR and western blot. As expected, the mRNA and protein levels of all these ER stress downstream molecules were significantly upregulated (Fig. 6F, G, Supplementary Fig. 9A). TUDCA is an ER stress inhibitor, which could significantly decrease the expression of several apoptosis associated critical factors, such as caspase-3 and caspase-12 [38, 39]. To confirm that activation of ER stress contributes to apoptosis in DNMT1-knockdown late stage erythroblasts, we examined the effects of TUDCA on cell growth and apoptosis. Notably, treatment with 100 μM TUDCA decreased the apoptosis from 22% to 13% (Fig. 6H, I) and increased cell growth (~3 fold) in DNMT1-deficient late erythroid cells (Fig. 6J). Our findings demonstrate that DNMT1 deficiency leads to apoptosis via excessive activation of ER stress pathway in late erythroblasts.

### DNMT1 deficiency leads to apoptosis via the p53-caspase-3 pathway in late terminal erythroblasts

**Fig. 7:**
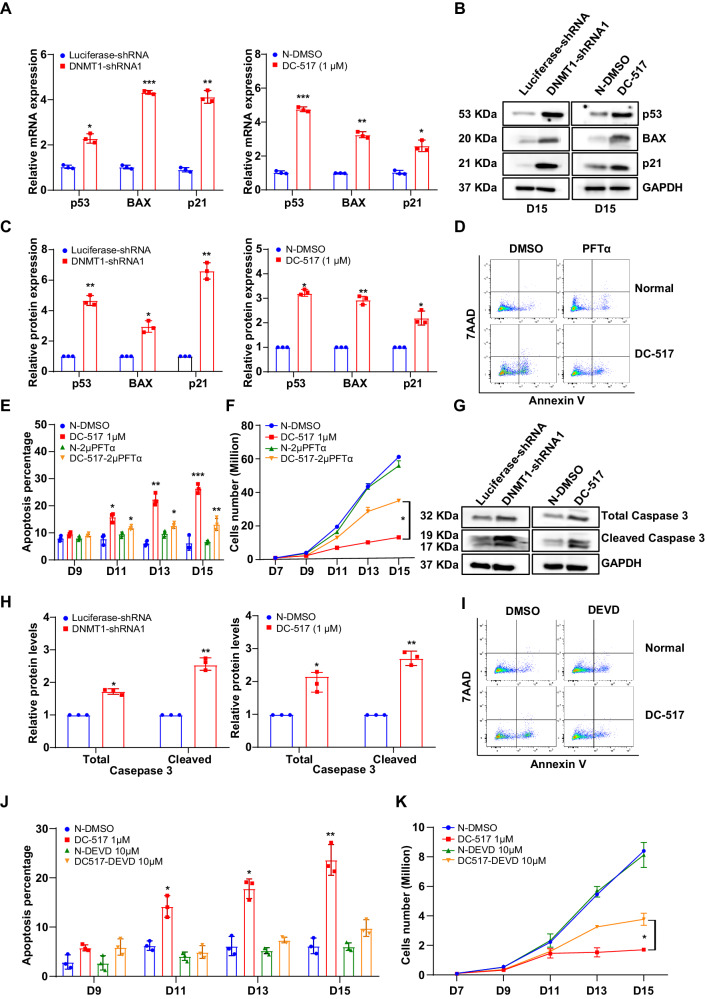
DNMT1 deficiency led to increased apoptosis via activation of p53-caspase-3 pathway. **A** qRT-PCR results showing p53, BAX, p21 mRNA expression levels in DNMT1 deficiency erythroblasts cultured for 15 days. GAPDH was used as internal reference. **B** Representative western blot analysis of p53, BAX, p21. GAPDH was used as loading control. **C** Quantitative analysis of p53, BAX, p21 from 3 independent experiments. **D** Representative flow cytometry profiles of apoptosis as assessed by Annexin V and 7AAD staining of Normal-DMSO and 1 μM DC_517 cells cultured for 15 days in the presence of DMSO or 2 μM PFTα. **E** Quantitative analysis of apoptosis from 3 independent experiments. **F** Growth curves of Normal-DMSO and 1 μM DC_517 cells in the presence of DMSO or 2 μM PFTα for 7, 9, 11, 13, and 15 days. **G** Representative western blot analysis of Total caspase-3, cleave caspase-3. GAPDH was used as loading control. **H** Quantitative analysis of caspase-3, cleave caspase-3 from 3 independent experiments. **I** Representative flow cytometry profiles of apoptosis as assessed by Annexin V and 7AAD staining of Normal-DMSO and DC_517 1 μM cells cultured for 15 days in the presence of DMSO or 10 μM DEVD. **J** Quantitative analysis of apoptosis from 3 independent experiments. **K** Growth curves of N-DMSO and 1 μM DC_517 cells in the presence of DMSO or 10 μM DEVD for 7, 9, 11, 13 and 15 days. * *p* < 0.05, ** *p* < 0.01, *** *p* < 0.001.

Cell death under ER stress depends on the core mitochondrial apoptosis pathway, which is mediated by the proapoptotic multidomain proteins BAX and the BH antagonist or killer (BAK) by triggering caspase activation [40]. p53 directly activates the proapoptotic Bcl-2 protein BAX in the absence of other proteins to permeabilize mitochondria and engage the apoptotic program [41]. Based on these findings, we hypothesize that p53-BAX is the downstream pathway of ER stress for regulating apoptosis. To test this thesis, we checked the expression of p53, BAX, and p21 by performing RNA-seq analysis and real-time PCR. As shown in Fig. 7A and Supplementary Fig. 9D, the mRNA levels of p53, BAX, and p21 were significantly upregulated in DNMT1-deficient late stage erythroblasts. Western blot analysis confirmed the increased protein levels of p53, BAX, and p21 in DNMT1-deficient cells (Fig. 7B, C). To examine whether the upregulation of p53 is associated with DNMT1 deficiency-mediated DNA methylation changes, we checked the methylation status of the *TP53* gene promoter at both early and terminal stage. We found that, at the early stage of erythropoiesis (day 6), methylation levels of the *TP53* promoter were 2.6% in the luciferase-shRNA group and 2.17% in the DNMT1 knockdown group. At the terminal stage of erythropoiesis (day 15), methylation levels of the *TP53* promoter were 2.17% in the luciferase-shRNA group and 1.74% in the DNMT1 knockdown group (Supplementary Fig. 10A, B). There was no significant difference in DNA methylation level of *TP53* promoter region between the control group and DNMT1 deficient group, indicating that DNMT1 mediated DNA methylation does not directly regulate p53 expression in both early and terminal stages of erythropoiesis. These findings further confirming our conclusion that the upregulation of p53 was caused directly by the excessive activation of ER stress, but not by the impairment DNA methylation induced by DNMT1 deficiency. To demonstrate that activation of the p53 pathway was responsible for DNMT1 deficiency-induced apoptosis, we examined the effects of the p53 inhibitor, PFTα, on cell growth and apoptosis. We found that treatment with PFTα not only promoted the cell growth, but also rescued the cell apoptosis of DNMT1-deficient cells (Fig. 7D–F). These findings indicate that the apoptosis of DNMT1-deficient cells was tightly associated with the upregulation and activation of p53-BAX pathway. Activation of caspase-3 have been well characterized as the final step for the p53-mediated apoptosis. To further explore the mechanism for the DNMT1 deficiency-induced apoptosis, we examined the protein expression of caspase-3 by western blot. DNMT1 deficiency caused significant upregulation of total caspase-3 and cleaved caspase-3 (Fig. 7G, H). Treatment with caspase-3 inhibitor, DEVD rescued the cell apoptosis of DNMT1-deficient late-stage erythroblasts with Annexin^+^ cells decreased from 30% to 10%, and restored cell growth with 2.5-fold increasing of the cell number (Fig. 7I–K). These findings demonstrate that the activation of the p53-caspase-3 pathway contribute to the impaired erythropoiesis caused by DNMT1 deficiency.

## Discussion

Dynamic regulation of DNA methylation and DNA demethylation is crucial for erythropoiesis [13–16]. Frequent changes in the stage-specific patterns of DNA methylation suggest that these dynamic regulatory elements are closely linked to the gene expression patterns that influence erythroid development and differentiation [17–20]. The roles of the Ten-eleven translocation (TET) methylcytosine dioxygenases (TET2, TET3), which are the key enzymes for the catalyzing of DNA demethylation, in the regulation of human erythropoiesis have been studied in depth by the works of our and other groups [13–15]. However, the roles of the DNA methyltransferase in the human erythropoiesis remains largely unknown. Our current study has revealed that DNMT1 plays a finely-tuned, stage-specific role in regulating human erythropoiesis. Specifically, it modulates the cell cycle during the early stage of erythroid development by inducing p21 methylation, ensuring normal cell cycle; while regulates terminal erythropoiesis by maintaining *RPL15* methylation to stabilize protein and ER stress homeostasis, thereby ensuring erythroid cell maturation and preventing apoptosis.

Our work discovered that DNMT1 deficiency caused cell cycle arrest by uniquely upregulating the expression of p21 in a p53 independent manner. Our findings are in line with previous research that showed DNMT inhibitor 5-Aza-2’deoxycytidine treatment induced cell cycle arrest in AML cells by inducing upregulation of p21 [42]. However, our study found that DNMT1 regulates p21 expression in erythroid progenitor cells by affecting the methylation of the promoter region of p21. Whereas, in AML cells, DNMT1 modulates p21 expression by regulating the methylation level of the p21 upstream gene, p73 [42]. Additionally, previous studies have shown that treatment with Decitabine, a pan-DNMT inhibitor, causes anemia by inducing DNA damage, cell cycle arrest, and apoptosis of the erythroid progenitor cells in patients with myelodysplastic syndrome and acute myeloid leukemia [43–46]. While, our research demonstrated that DNMT1-deficient erythroid progenitors exhibited only cell cycle arrest, rather than apoptosis. This could be attributed to the strategy we used in the current work, by which we specifically knocked down or inhibited DNMT1, but not other members of DNMT family. Whereas, as a pan inhibitor, treatment with Decitabine will lead to the dysfunctionality of all the members of DNMTs [47, 48]. Taken into consideration that DNMT3A and DNMT3B were expressed at a relatively high levels during early stage, the findings from us and others strongly suggest that DNMT1 might regulate early-stage human erythropoiesis in cooperation of DNMT3A and DNMT3B. In addition, our findings provide insights into the anemia caused by DNA demethylation agent treatment.

It is interesting to note that, although DNMT1 deficiency also resulted in the upregulation of p21 expression in terminal-stage erythroblasts, treatment with p21 inhibitor did not rescue DNMT1 deficiency-induced apoptosis of erythroblasts. Alternatively, partial restoration of the cell apoptosis was achieved with the treatment with p53 or caspase-3 inhibitor. We speculate that the cell cycle exit, which is considered as one of the hallmark biological events during the terminal stage, is responsible for the phenomenon that p21 is no longer involved in regulating cell expansion in the terminal stage. Therefore, we conclude that DNMT1 deficiency-caused impairment of cell expansion in not mediated by the p21-dependent cell cycle block. Thus, our findings not only confirm earlier reports that p21 activation impairs erythropoiesis in the early stage of erythropoiesis [49], but also provide further confirmation for the stage-specific roles of DNMT1 in the regulation of erythropoiesis.

Despite erythroid cells showing a global trend of DNA demethylation in the progression of erythroid development [17], we found that DNMT1 was still highly expressed in the late stage of erythroid terminal differentiation. Our current work revealed that DNMT1 deficiency could trigger an overall increase in the transcription level of core RPs by upregulating the expression of RPL15 which was due to decreased methylation level of the RPL15 promoter region, thereby accelerating protein synthesis [33, 35]. Global high-level expression of RPs will inevitably accelerate translation speed, which we have confirmed through O-propargyl-puromycin experiments [33, 35, 50, 51]. The accelerated protein load in the ER activates all branches of the UPR, leading to apoptosis in terminal late erythroblasts [32–35]. The ER stress is an intracellular adaptive regulatory biological process characterized by the accumulation of misfolded and unfolded proteins [52–54]. On one hand, DNMT1 deficiency re-activated the expression of genes suppressed by DNA methylation, and on the other hand, it mediates the overall expression of core RPs, with RPL15 acting as the central hub. This results in an accelerated rate of intracellular protein translation and sustained ER stress. The cell fails to restore ER homeostasis, ultimately activating the apoptotic pathway and triggering cell death. Remarkably, dysfunctional ER stress have been reported in β-thalassemia patients [55]. Furthermore, reduced level of DNMT1 protein has been observed in patients with β-thalassemia [22]. These findings provide additional evidence that DNMT1 deficiency may induce apoptosis through the activation of ER stress. Surprisingly, ER stress inhibitor could reverse the apoptosis of DNMT1-deficient erythroblasts in the terminal erythroblasts, indicating that activation of ER stress can lead to apoptosis in normal erythroblasts and that ER stress inhibitor could be a potential protective approach for erythroblasts in patients undergoing DNMT1 inhibitor treatment. Our findings shed light on the other molecular roles of DNMT1 in erythroblasts and suggest a potential avenue for mitigating the severity of β-thalassemia through DNMT1 deficiency.

To summarize, our research has revealed previously undiscovered mechanistic roles for DNMT1 in the regulation of human erythropoiesis. These findings offer new insights into the function of DNMT1 in regulating erythropoiesis.

Note: Supplementary information is available at Cell Death & Differentiation website.

### Supplementary information

Supplementary Data

Supplementary Data-Original western blot

## Data Availability

Data and materials supporting the findings are available from the corresponding authors upon request.
