# Supplementary material for: DNMT1 regulates human erythropoiesis by modulating cell cycle and endoplasmic reticulum stress in a stage-specific manner
Source: Cell Death Differ. 2024 May 8;31(8):999–1012. doi: 10.1038/s41418-024-01305-6 (PMC11303534; doi:10.1038/s41418-024-01305-6)
Supplement: Supplementary file 1 — Supplementary Data [file 41418_2024_1305_MOESM1_ESM.docx]

**Supplementary materials and methods**

**Antibodies**

Antibodies used for western blott were as follows: rabbit anti-human DNMT1 (cat. no. A5495, ABclonal), rabbit anti-human glyceraldehyde-3-phosphate dehydrogenase GAPDH (cat. no. AB2000, ABways), mouse anti-human caspase-3 (cat. no. 66470-2-Ig, Proteintech), mouse anti-p53 (cat. no. 60283-2-Ig, Proteintech), mouse anti-p21 (cat. no. 60214-1-Ig, Proteintech), rabbit anti-RPL15 (cat. no.16470-1-AP, Proteintech), mouse anti-BAX (cat. no. 60267-1-Ig, Proteintech), rabbit anti-human activating transcription factor 4 ATF4 (cat. no. 10835-1-AP, Proteintech), rabbit anti-human protein kinase R-like endoplasmic reticulum kinase PERK (cat. no. 5683T, Cell Signaling Technology), rabbit anti-human inositol-requiring transmembrane kinase endoribonuclease-1α IRE1α (cat. no. 3294T, Cell Signaling Technology), rabbit anti-human binding immunoglobulin protein BIP (cat. no. 3177, Cell Signaling Technology), rabbit anti-human eukaryotic initiation factor 2 eIF2α (cat. no. 5324T, Cell Signaling Technology), rabbit anti-human Phospho-eIF2α (Ser51) p-eIF2α (cat. no. 3398T, Cell Signaling Technology), mouse anti-human C/EBP homologous protein CHOP (cat. no. 2895T, Cell Signaling Technology), rabbit anti-human Phospho-IRE1α (S724) p-IRE1α (cat. no. ab124945, abcam). The antibody used for dot blotting was mouse anti-human 5-Methylcytosine 5-mC (cat. no. 61255, ACTIV&MOTIF). Commercial antibodies used for flow cytometry were as follows: APC-conjugated CD235a (GPA) (cat. no. 551336, BD), PE-conjugated CD235a (GPA) (cat. no. 8065789, BD), PE-conjugated CD34 (cat. no. 555822, BD), PE-conjugated CD49d (α4 integrin) (cat. no. 12-499-42, invitrogen), APC-conjugated CD49d (α4 integrin) (cat. no. 130-124-229, MACS), FITC-conjugated CD36-fluoresce (cat. no. 555454, BD), PE-cy7- conjugated CD123 (IL-3R) (cat. no. 25-1239-42, invitrogen), APC-conjugated annexin V (cat. no. 88-8103-74, eBioscience), FITC-conjugated annexin V (cat. no. 640945, Biolegend) Hoechst33342 (cat. no. C0031, Solarbio), and 7AAD (cat. no. 00-6993-50, eBioscience). Mouse monoclonal antibody against human band 3 was generated in our laboratory and labeled with FITC or APC as described previously [1].

Culture of CD34^+^cells
CD34^+^ cells were differentiated to erythroid lineages as described previously [1,2]. The details were as follows: The day of getting CD34^+^ cells were recorded as day 0. The cell culture procedure was comprised of 3 phases. Composition of the base culture medium was Iscove’s Modified Dulbecco’s Medium, 2% human peripheral blood plasma, 3% human AB serum, 200 mg/mL human Holo-transferrin, 3 IU/mL heparin, and 10 mg/mL insulin. In the first phase (day 0 to day 7), CD34^+^ cells at a concentration of 10^5^ /mL were cultured in the presence of 10 ng/mL stem cell factor, 1 ng/mL IL-3, and 3 IU/mL erythropoietin. In the second phase (day 7 to day 11), IL-3 was omitted. In the third phase that lasted until day 21, the cell concentration was adjusted to 10^6^ /mL on day 11 and to 5×10^6^/mL on day 15, respectively, the medium for this phase was the base medium plus 3 IU/mL erythropoietin, and the concentration of transferrin was adjusted to 1 mg/mL.

**Cell cycle analysis**

Edu kit was used for cell cycle measurement according to the manufacturer’s protocol. In brief, 1 × 10^6^ cells were incubated with 10 μM Edu for 2 h at 37 °C. After incubation, the cells were harvested and washed with 3 ml of 1% BSA in PBS. Cells were then fixed, permeabilized, and stained with Edu detection cocktail as well as 7AAD. The staining of Edu and 7AAD was analyzed by flow cytometry. Data were collected and analyzed using FlowJ, and the data are expressed as EdU fluorescence intensity versus 7AAD [3].

**RNA extraction and** **quantitative reverse transcription-PCR assays**

RNA was extracted from cell cultures using RNA extract kits (cat. no. 74104, Qiagen) according to the manufacturer’s instructions. Reverse transcription was performed using a HiFi-MMLV cDNA Kit (cat. no. CW0744M, CWBIO). Quantitative reverse transcription PCR (qRT-PCR) assays were completed using SYBR™ Select Master Mix (cat. no. 4472903, Thermo Fisher Scientific). Quantitative reverse transcription-PCR was carried out on a PCR platform (LightCycler 480, Roche Life Science) using a master mix according to the manufacturer’s instructions. Relative expression levels were normalized to that of GAPDH. The primer sequences are listed in Supplemental Table 1. Detailed protocols for the RNA extraction, reverse transcription, primer design, and quantitative reverse transcription-PCR have been described previously [4].

**Western blot**

Total cell lysates were prepared using RIPA buffer (cat. no. 89900, Thermo Fisher Scientific) in the presence of proteinase inhibitor Cocktail (cat. no. P8340, Sigma). Protein concentration was measured using a BCA protein assay kit (cat. no. P1511-5, PPLYGEN). All procedures were performed as described previously [4].

**Culture HUDEP-2**

HUDEP-2 cells were cultured in expansion medium: HUDEP-2 cells were cultured in expansion medium: StemSpan Serum-Free Expansion Medium (SFEM, cat. no. 09650, Stemcell Technologies), 2% Penicillin Streptomycin solution (10,000 U/mL stock), 50 ng/mL recombinant human stem cell factor (SCF), 3 IU/mL Epoetin alfa, 0.4 μg/mL dexamethasone, 1 μg/mL doxycycline. To induce differentiation, HUDEP-2 cells were cultured in IMDM supplemented with FBS (10%), 5% plasma, 1 μg/mL doxycycline, EPO (3 IU/mL), transferrin 330µg/mL, hSCF (100 ng/mL), and L-glutamine1% for 5 days and then transferred them into IMDM supplemented with FBS (10%), 5% plasma, 1 μg/mL doxycycline, EPO (3 IU/mL), transferrin 330µg/mL, and L-glutamine1% for 5days.

**Genome editing by CRISPR/Cas9**

CRISPR/Cas based gene-editing strategy were used to generate DNMT1 knockout HUDEP-2 cells. sgRNAs was designed using the online gRNA design tool (https://bioinfogp.cnb.csic.es/tools/breakingcas/index.php.sgRNA). The sequence of sgRNA are listed in Supplemental Table 3. Oligonucleotides were cloned into the lenti-CRISPR vector through restriction site BsmBI (cat. no. R0739s, NEB). Lentiviral transduction was performed as described below. The medium used for transfection：StemSpan Serum-Free Expansion Medium (SFEM, Stemcell Technologies), 50 ng/mL recombinant human stem cell factor (SCF), 3 IU/mL EPO, 0.4 μg/mL dexamethasone, 1 μg/mL doxycycline, 8μg/mL polybrene. Transfection was performed according to the cell density of 0.5 × 10^6^/ml for 12-16 h. After transfection, the medium was changed to HUDEP-2 expansion medium, and purinomycin 1mg/mL was added 24 hours later for screening. Fluorescence-activated cell sorting was then performed to collect DNMT1 knockout HUDEP-2 cell.

**CRISPRi/dCas9-mediated knockdown**

dCas9-KRAB CRISPRi based gene-editing system was used to generate DNMT1 silenced CD34^+^ HSPCs. dsgRNAs were designed using the online tool (<https://portals.broadinstitute>.org/gppx/crispick public). The sequence of dsgRNA are listed in Supplemental Table 3. Oligonucleotides were cloned into the pLV hU6-sgRNA hUbC-dCas9-KRAB-T2a-Puro vector through restriction site BsmBI. Lentiviral transfection was performed according to the strategy of described above. CD34^+^ cells were differentiated towards the erythroid lineage using a three-phase culture system that have been described previously [3,5].

**REFERENCES**

1. Li J, Hale J, Bhagia P, Xue F, Chen L, Jaffray J, et al. Isolation and transcriptome analyses of human erythroid progenitors: BFU-E and CFU-E. Blood. 2014;124(24):3636-45.
2. Hu J, Liu J, Xue F, Halverson G, Reid M, Guo A, et al. Isolation and functional characterization of human erythroblasts at distinct stages: implications for understanding of normal and disordered erythropoiesis in vivo. Blood. 2013;121(16):3246-53.
3. Huang Y, Hale J, Wang Y, Li W, Zhang S, Zhang J, et al. SF3B1 deficiency impairs human erythropoiesis via activation of p53 pathway: implications for understanding of ineffective erythropoiesis in MDS. J Hematol Oncol. 2018;11(1):19.
4. Qu X, Zhang S, Wang S, Wang Y, Li W, Huang Y, et al. TET2 deficiency leads to stem cell factor-dependent clonal expansion of dysfunctional erythroid progenitors. Blood. 2018 Nov 29;132(22):2406-2417.
5. Yan H, Wang Y, Qu X, Li J, Hale J, Huang Y, et al. Distinct roles for TET family proteins in regulating human erythropoiesis. Blood. 2017;129(14):2002-12.

**Supplementary Figures**

**Supplemental Fig 1. dCas9 mediated DNMT1 knockdown caused cell cycle arrest at G1 phase of erythroid progenitors.**

(A) qRT-PCR results showing DNMT1 mRNA expression levels in Control or dCas9-DNMT1-dsgRNA transduced erythroblasts at day 6. GAPDH was used as internal reference. (B) Representative flow cytometry profiles of cell cycle as assessed by Edu and 7AAD staining of cells cultured at day 6. (C) Quantitative analysis of cell cycle from 3 independent experiments. (D) Representative flow cytometry profiles of apoptosis as assessed by dual staining of Annexin V^+^ and 7AAD of cells cultured for Day6. (E) Quantitative analysis of apoptosis from 3 independent experiments. ** *p*< 0.01, *** *p*<0.001.

**Supplemental Fig 2. Cas9 mediated DNMT1 knockout leads to significant reduction of cell expansion by inducing cell cycle arrest at G1 phase of HUDEP-2 cells cultured in expansion medium.**

(A) Representative western blot showing protein expression levels DNMT1 in Control and Cas9-DNMT1-sgRNA transduced HUDEP-2 at day 0. (B) Quantitative analysis of DNMT1 protein levels from 3 independent experiments. (C) Growth curves of cells of control group or Cas9-DNMT1-sgRNA transduced cells at 0, 2, 4, 6 and 8 days, respectively. (D) Representative flow cytometry profiles of cell cycle as assessed by Edu and 7AAD staining of cells cultured at day 0. (E) Quantitative analysis of cell cycle from 3 independent experiments. ** *p*< 0.01, *** *p*<0.001.

**Supplemental Fig 3. Deficiency of DNMT1 mediated by Cas9 and dCas9 strategy leads to an increase in apoptosis in terminal erythroblasts.**

(A) Representative flow cytometry profiles of apoptosis as assessed by dual staining of Annexin V+ and 7AAD of HUDEP-2 cells induced for 0, 2, 4, 6, and 8 days, and quantitative analysis of apoptosis from 3 independent experiments. (B) Representative flow cytometry profiles of apoptosis as assessed by dual staining of Annexin V^+^ and 7AAD of CD34^+^ cells induced for 7, 9, 11, 13, and 15 days, and quantitative analysis of apoptosis from 3 independent experiments. * *p*< 0.05, ** p< 0.01, *** p<0.001.

**Supplemental Fig 4. DNMT1 deficiency showed no significant effects on early erythroid differentiation.** (A) Representative flow cytometry profiles of cell differentiation of erythroid progenitors. Cells cultured for 6 days were stained with antibodies against IL-3R, GPA, CD34 and CD36. The IL-3R and GPA double-negative population was further separated by CD34 and CD36. The IL-3R^−^GPA^−^CD34^+^CD36^−^ population was identified as BFU-E and the IL-3R^−^GPA^−^CD34^−^CD36^+^ population was identified as CFU-E. (B) Quantification of BFUE and CFU-E at day6. **p* < 0.05; ***p* < 0.01.

**Supplemental Fig 5. DNMT1 deficiency slightly delayed terminal erythroid differentiation.**

(A) Representative flow cytometry profiles of the expression of GPA of erythroblast at day 7 and day 15. (B) Quantitative analysis of GPA^+^ positive cells at day 7, 11 and 15. (C) Terminal erythroid differentiation was monitored at day 7 and day 15 using flow cytometric analysis based on the expression of band 3 and α4 integrin. Representative plots of α4-integrin vs band 3 of GPA^+^ cells are shown and the erythroblasts are separated into 5 populations: proerythroblasts (I), early basophilic erythroblasts (II), late basophilic erythroblasts (III), polychromatic erythroblasts (IV), and orthochromatic erythroblasts (V). (D) Quantitative analyses of erythroblasts at distinct stages. (E) Representative cytospin images of erythroblasts on 7, 9, 11, 13 and 15 days stained with May-Grünwald Giemsa. Scale bar = 10μm. The results are from 3 independent experiments. **p* < 0.05; ***p* < 0.01, *** *p*<0.001.

**Supplemental Fig 6. DNMT1 regulate *p21* methylation level in erythroid progenitors.**

(A) Representative DNA dot blots showing 5mC level of at day6 in the shRNA mediated DNMT1 knockdown erythroid progenitors. (B) Quantitative analysis of 5mC levels from 3 independent experiments. (C) Representative DNA dot blots showing 5mC level of at day 0 of Cas9 mediated DNMT1 knockout HUDEP2 cells cultured in expansion medium. (D) Quantitative analysis of 5mC levels from 3 independent experiments. (E) Prediction of *p21* promoter regions CPG island. (F) Pyrosequencing result showed there were 28 CpG sites of *p21* promoter region named pos1-pos28. (G) Quantitative analysis of the methylation status *p21* CpG sties in control and DNMT1-shRNA knock down group at day 6. * *p*< 0.05.

**Supplemental Fig 7. DNMT1 regulated *RPL15* promoter regions methylation level in late stage of terminal erythroblasts.**

(A) Representative DNA dot blots showing at day 15 in the shRNA mediated DNMT1 knock out terminal erythroblast. (B) Quantitative analysis of 5mC levels from 3 independent experiments. (C) Representative DNA dot blots showing 5mC level of at day 8 of Cas9 mediated DNMT1 knockout HUDEP2 cells cultured in differentiation medium. (D) Quantitative analysis of 5mC levels from 3 independent experiments. (E) Prediction of *RPL15* promoter regions CPG island. ** *p*< 0.01.

**Supplemental Fig 8. DNMT1 deficiency leads to increased apoptosis of late erythroblasts in a p21 independent manner.**

(A) Representative flow cytometry profiles of apoptosis as assessed by Annexin V and 7AAD staining of luciferase-shRNA and DNMT1-shRNA cells cultured for 15 days in the presence of DMSO or 1μM UC2288. (B) Quantitative analysis of apoptosis from 3 independent experiments. (C) Representative flow cytometry profiles of apoptosis as assessed by Annexin V and 7AAD staining of Normal-DMSO and DC_517 1μM cells cultured for 15 days in the presence of DMSO or 1μM UC2288. (D) Quantitative analysis of apoptosis from 3 independent experiments. (E) Bisulfite sequencing analysis of methylation profile of *p21* promotor region at day 15. * *p*< 0.05, ** *p*< 0.01.

**Supplemental Fig 9. The mRNA expression level of the genes encoded p53 pathway and ER stress related molecules.**

(A) qRT-PCR results showing mRNA levels of ER stress related genes, including *ATF4, XBP1*, *DDIT3* in DNMT1 deficient terminal erythroblast cultured for 15 days. (B,C) qRT-PCR results showing *ATF4, XBP1*, *DDIT3* and BIP mRNA expression levels in DNMT1 deficiency early stage erythroid progenitors cultured for 6 days. GAPDH was used as internal reference. (D) qRT-PCR results showing mRNA levels of p53 pathway related genes, including *TP53, BAX* and *p21* in DNMT1 deficient terminal erythroblast cultured for 15 days. * *p*< 0.05, ** *p*< 0.01, *** *p*<0.001.

**Supplemental Fig 10. DNMT1 deficiency showed no effect on the methylation of *TP53* promoter region.**

(A、B) Bisulfite sequencing analysis of CpG islands in *TP53* promoter region at day 6 and day 15. Methylated (black circle) and unmethylated (white circle) CpG dinucleotides are displayed. Shown here are the percentages of methylation in DNMT1-deficient cell and normal erythroid cells.


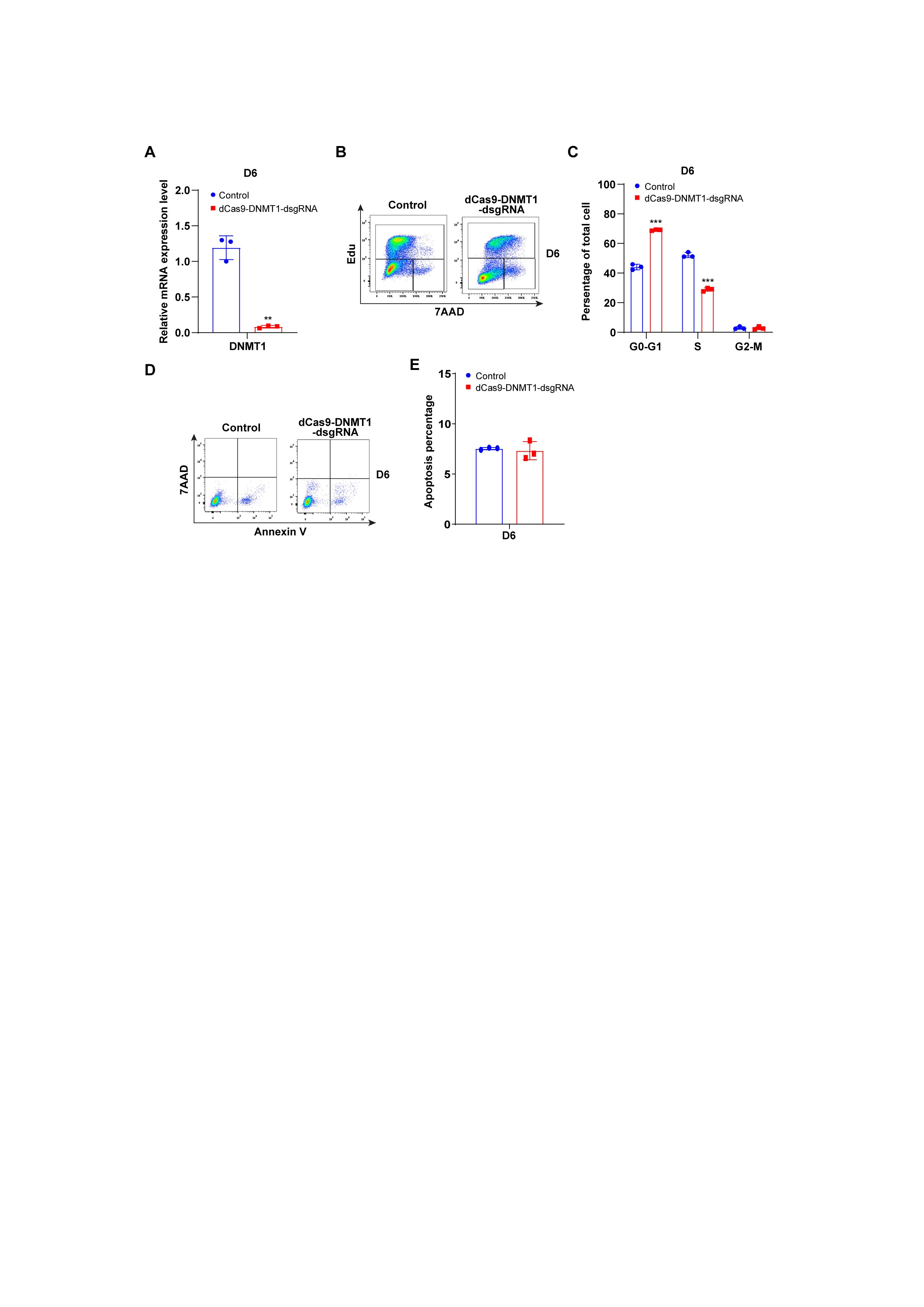


**Supplemental Fig 1.** dCas9 mediated DNMT1 knockdown caused cell cycle arrest at G1 phase of erythroid progenitors.


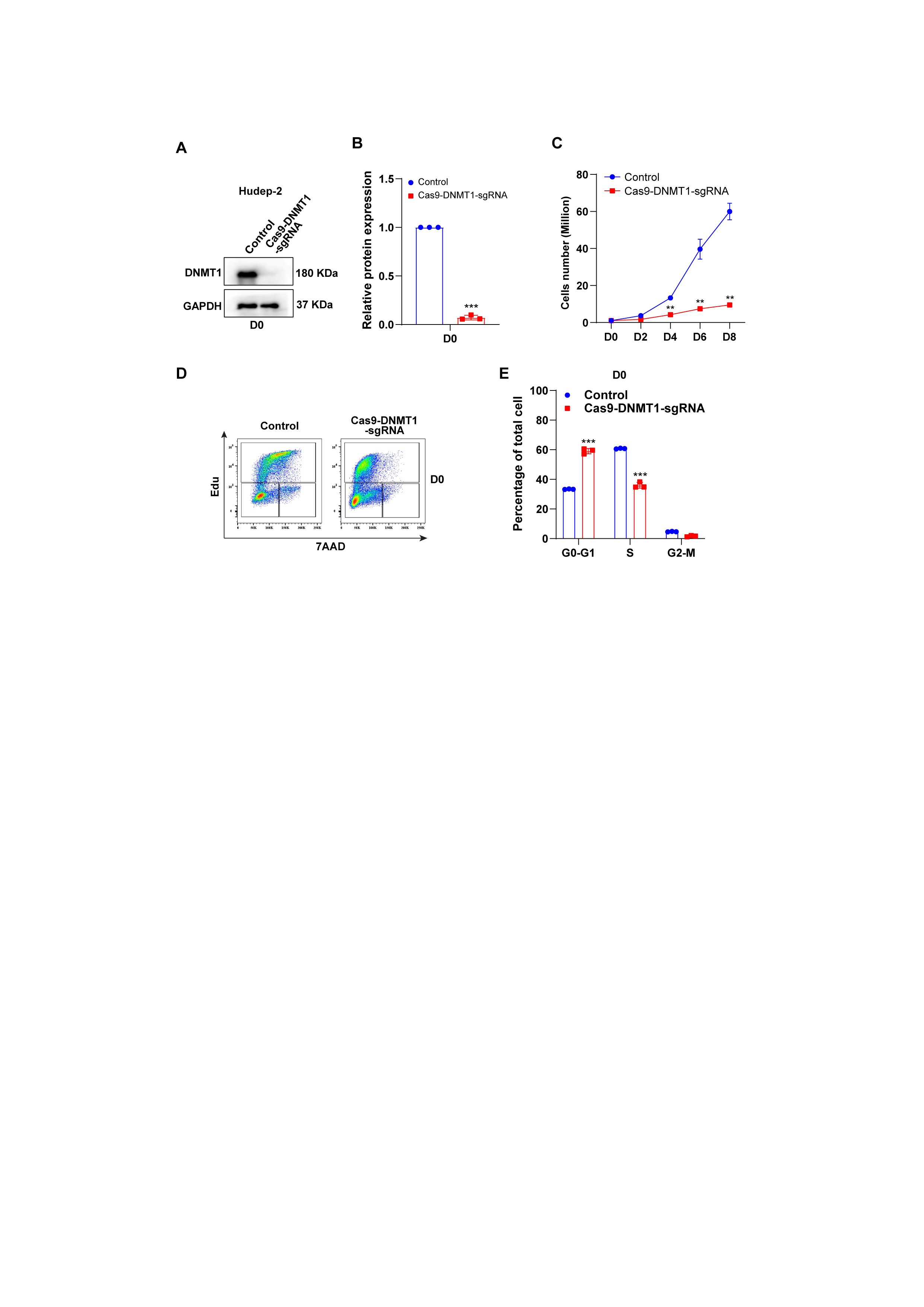


**Supplemental Fig 2.** Cas9 mediated DNMT1 knockout leads to significant reduction of cell expansion by inducing cell cycle arrest at G1 phase of HUDEP-2 cells cultured under expansion medium.


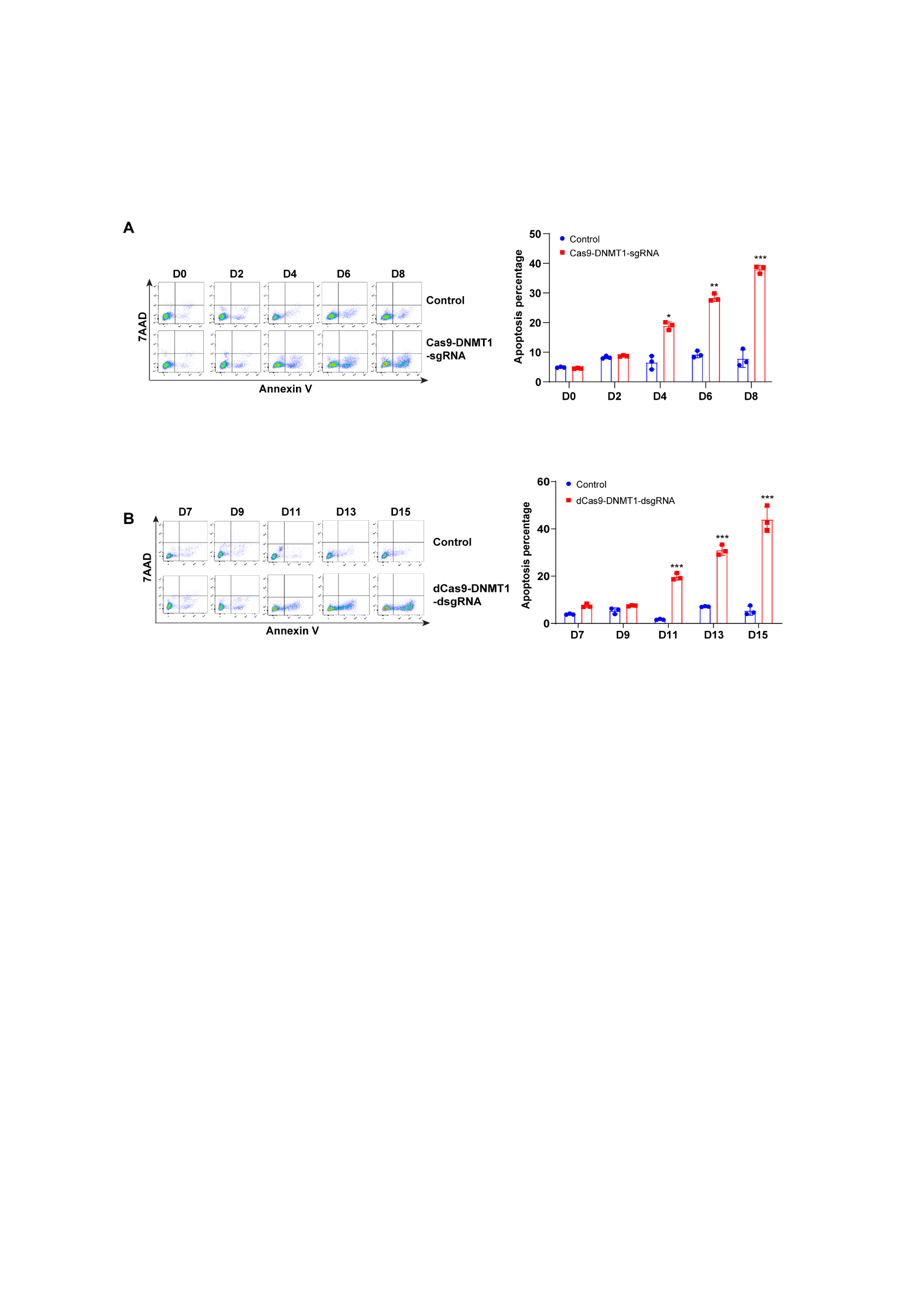


**Supplemental Fig 3.** Deficiency of DNMT1 mediated by Cas9 and dCas9 strategy leads to an increase in apoptosis in terminal erythroblasts.


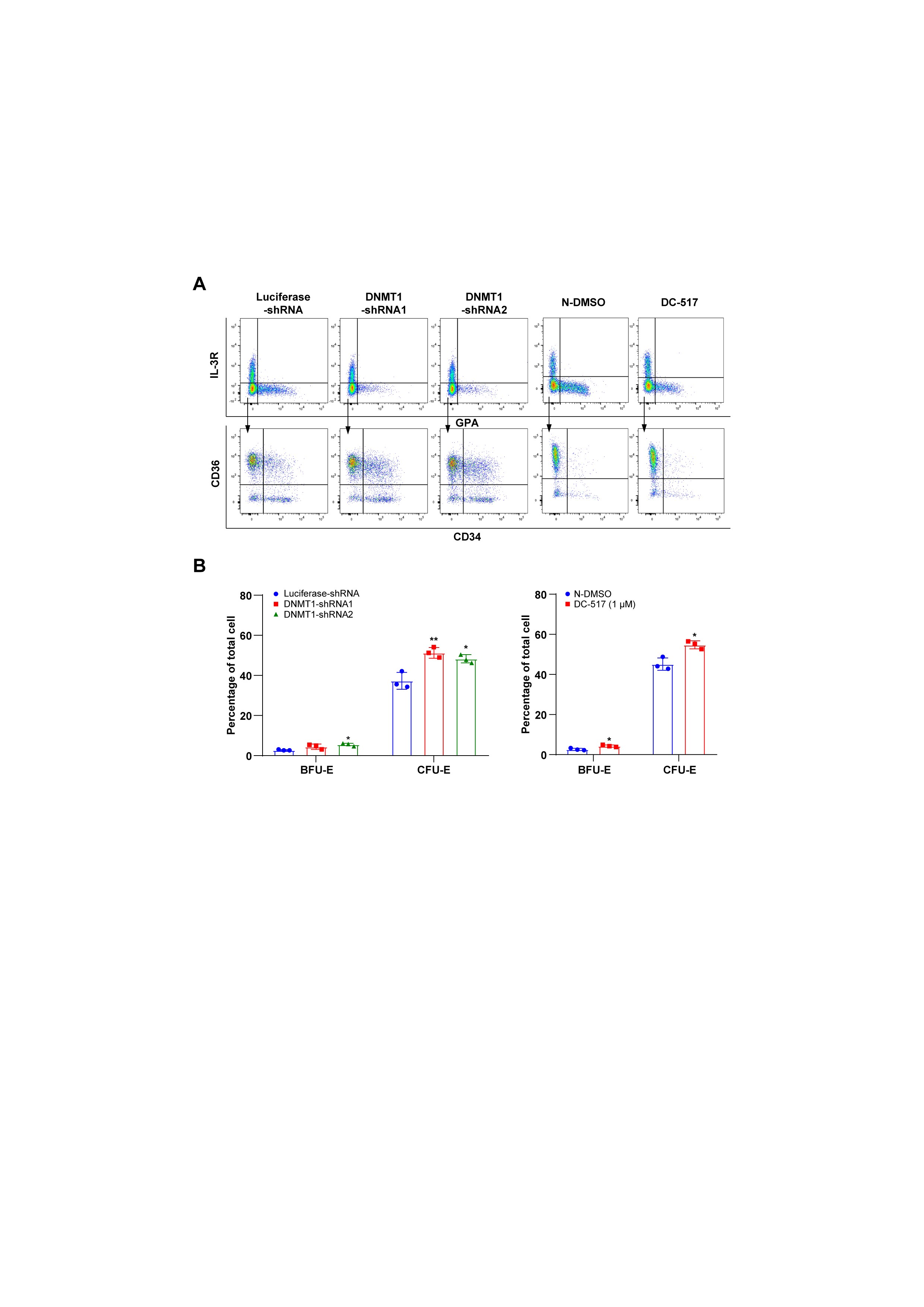


**Supplemental Fig 4.** DNMT1 deficiency showed no significant effects on early erythroid differentiation.


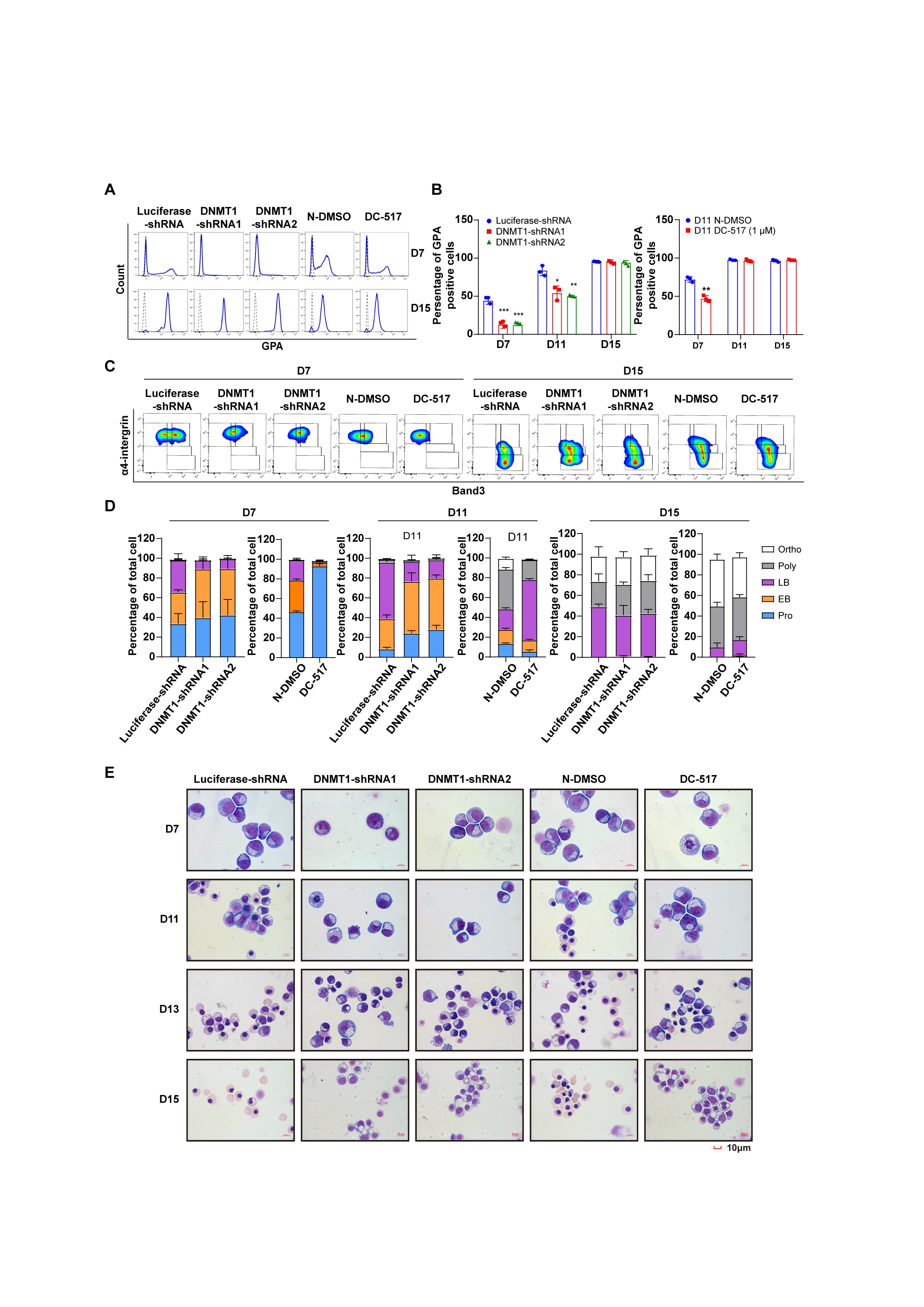


**Supplemental Fig 5.** DNMT1 deficiency slightly delayed terminal erythroid differentiation


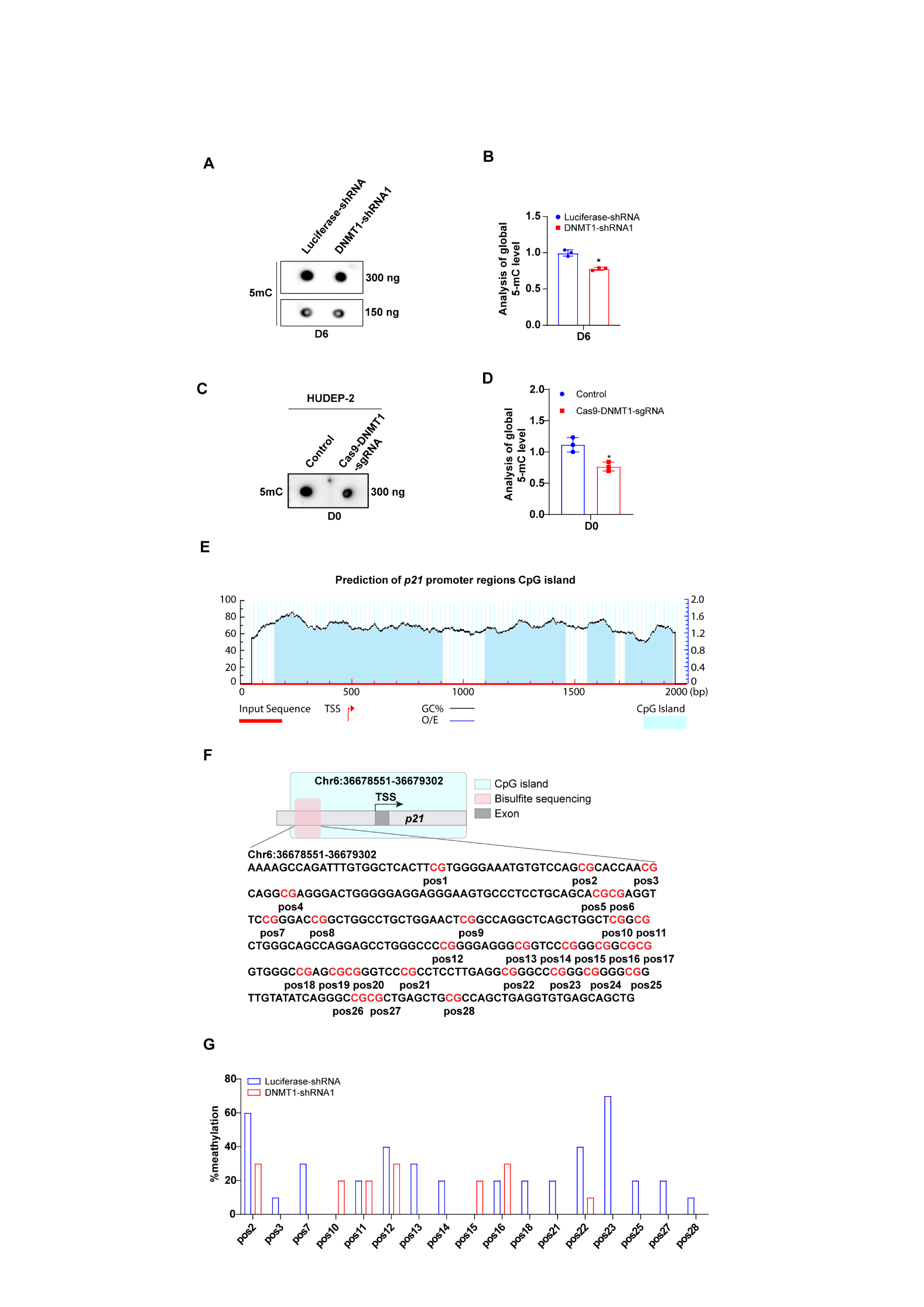


**Supplemental Fig 6.** DNMT1 regulate p21 methylation level in erythroid progenitors.


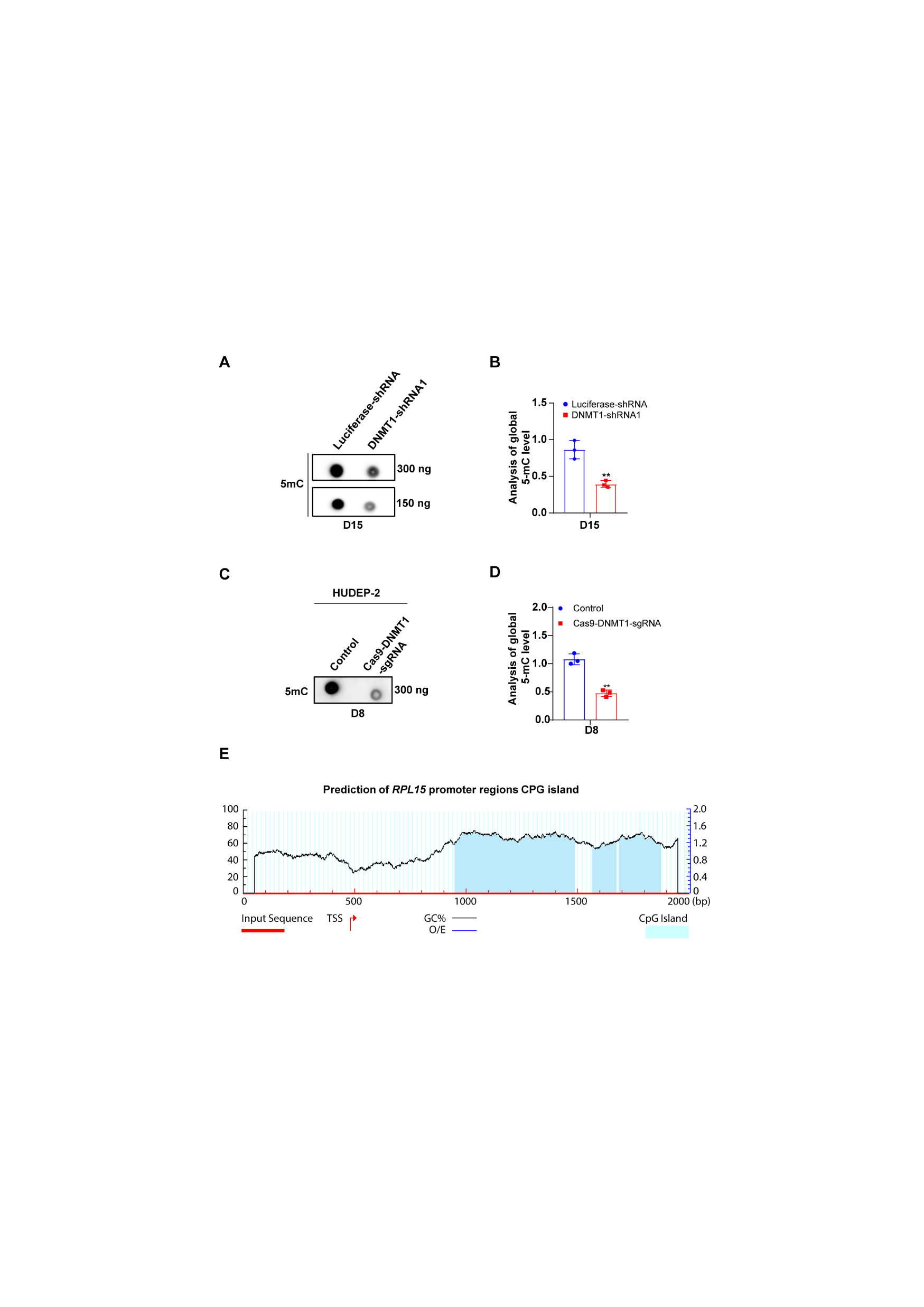


**Supplemental Fig 7.** DNMT1 regulated *RPL15* promoter regions methylation level in late stage of terminal erythroblasts.


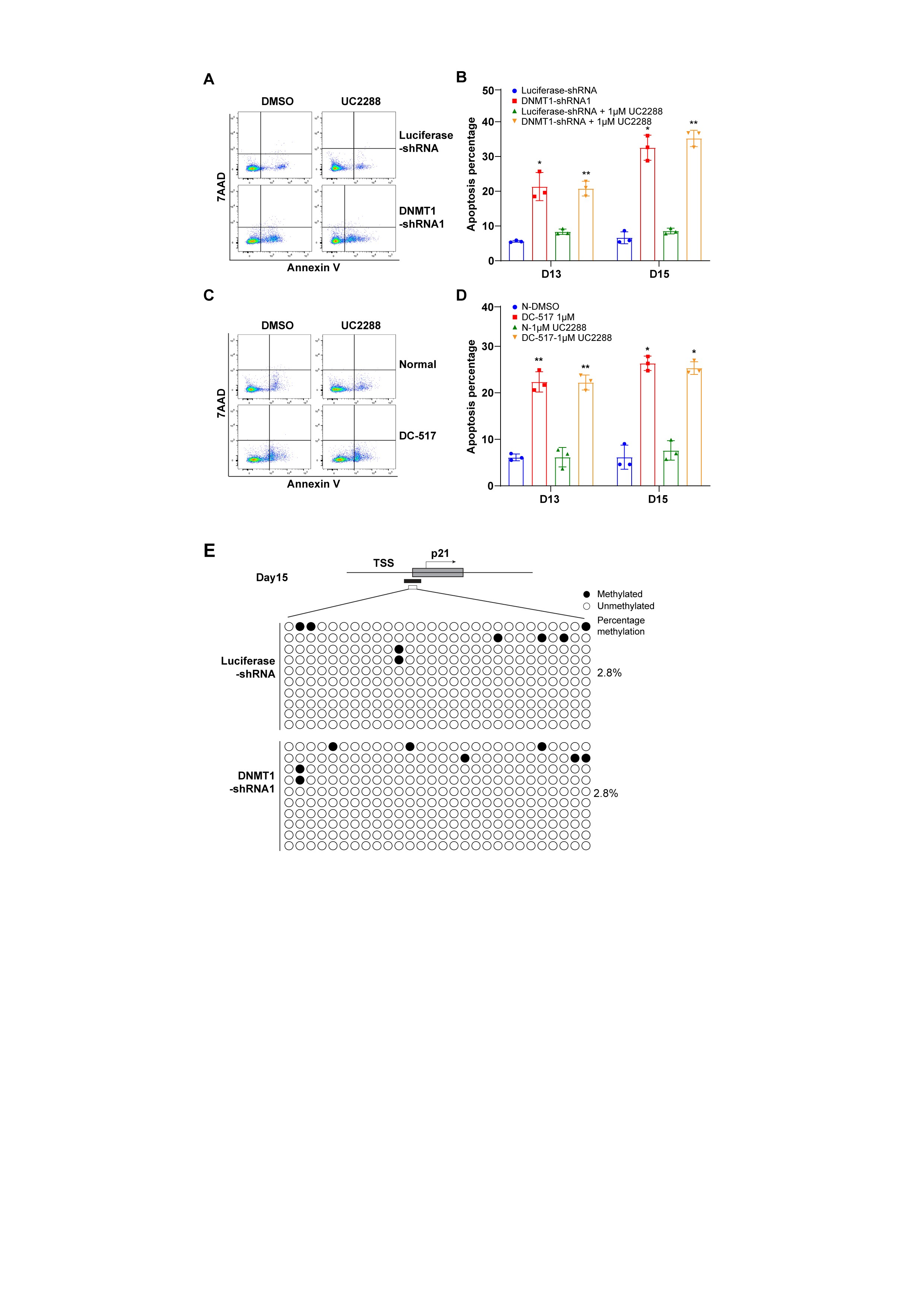


**Supplemental Fig 8.** DNMT1 deficiency leads to increased apoptosis of late erythroblasts in a p21 independent manner.


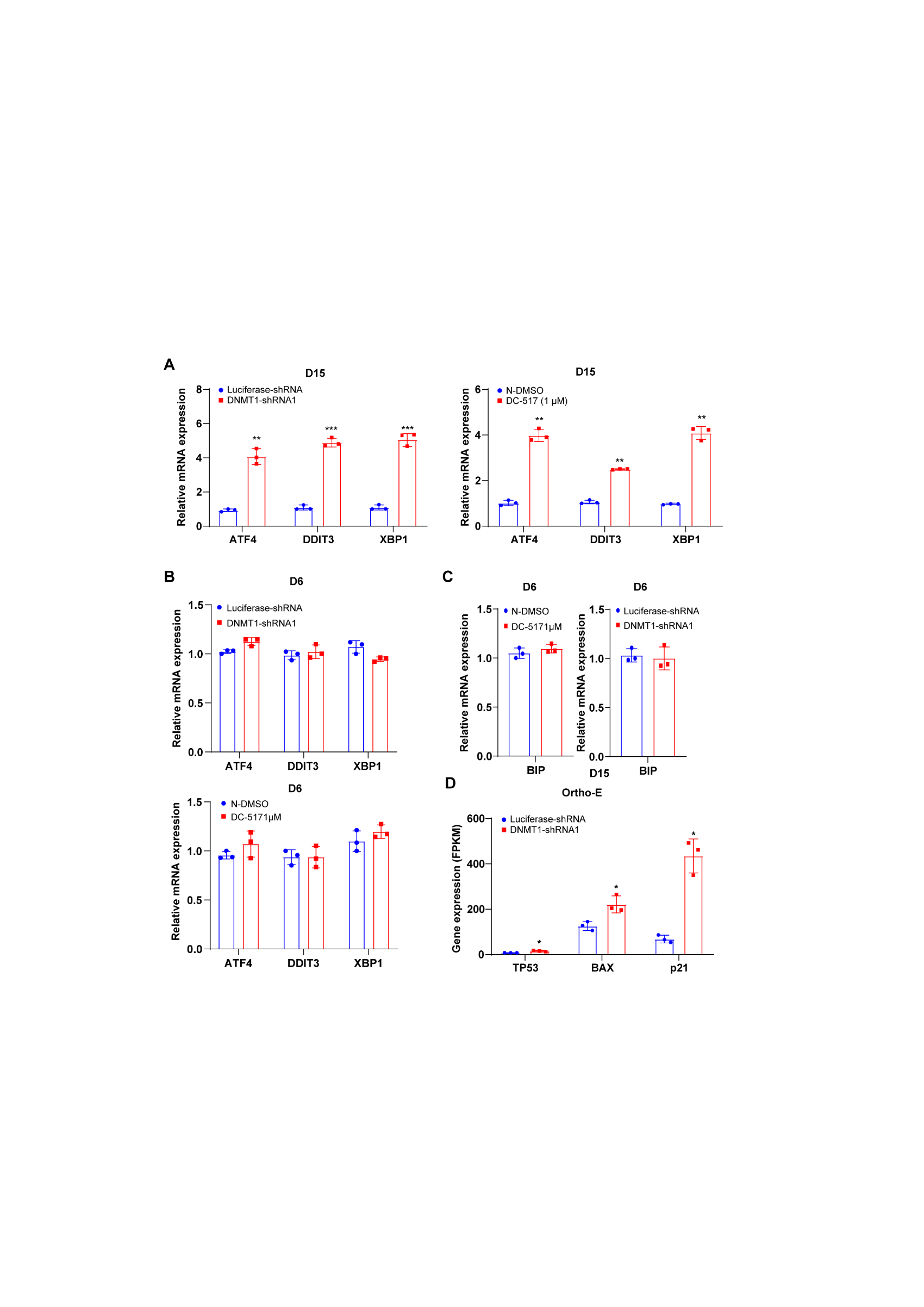


**Supplemental Fig 9.** The mRNA expression level of the genes encoded p53 pathway and ER stress related molecules.


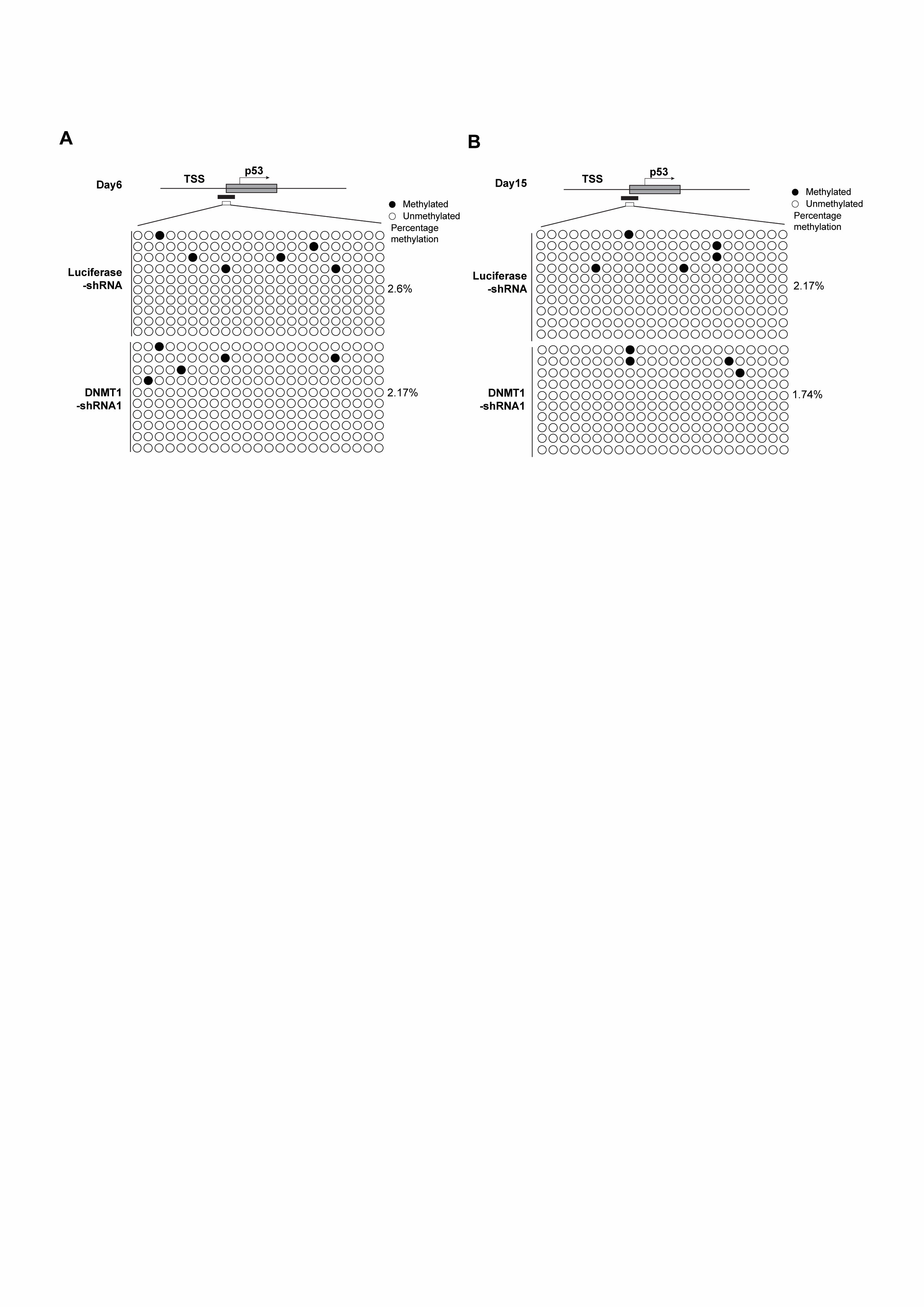


**Supplemental Fig 10.** DNMT1 deficiency showed no effect on the methylation of *TP53* promoter region.

| **Gene** | **Sequence** | |
| --- | --- | --- |
| DNMT1 | Forward | 5'- CCTAGCCCCAGGATTACAAGG -3' |
|  | Reverse | 5'- ACTCATCCGATTTGGCTCTTTC -3' |
| GAPDH | Forward | 5'- CATGAGAAGTATGACAACAGCCT -3' |
|  | Reverse | 5'- AGTCCTTCCACGATACCAAAGT -3' |
| p21 | Forward | 5'- TGTCCGTCAGAACCCATGC-3' |
|  | Reverse | 5'- AAAGTCGAAGTTCCATCGCTC-3' |
| TP53 | Forward | 5'- TGTTTCCTGACTCAGAGGGG-3' |
|  | Reverse | 5'-GAGCGTGCTTTCCACGAC-3' |
| BAX | Forward | 5'-TGCCTCAGGATGCGTCCACCAA-3' |
|  | Reverse | 5'-CCCCAGTTGAAGTTGCCGTCAG-3' |
| ATF4 | Forward | 5'-CTCCGGGACAGATTGGATGTT-3' |
|  | Reverse | 5'-GGCTGCTTATTAGTCTCCTGGAC-3' |
| DDIT3 | Forward | 5'-GGAAACAGAGTGGTCATTCCC-3' |
|  | Reverse | 5'-CTGCTTGAGCCGTTCATTCTC-3' |
| BIP | Forward | 5'-GAAAGAAGGTTACCCATGCAGT-3' |
|  | Reverse | 5'-CAGGCCATAAGCAATAGCAGC-3' |
| XBP1 | Forward | 5'-CCCTCCAGAACATCTCCCCAT-3' |
|  | Reverse | 5'-ACATGACTGGGTCCAAGTTGT-3' |
| p16 | Forward | 5'-GGGTTTTCGTGGTTCACATCC-3' |
|  | Reverse | 5'-CTAGACGCTGGCTCCTCAGTA-3' |
| p15 | Forward | 5'-AAGTCTGGCTCGTTCTCAGTG-3' |
|  | Reverse | 5'-TGGACCTGGTGGCTACGAAT-3' |
| p57 | Forward | 5'-GCGGCGATCAAGAAGCTGT-3' |
|  | Reverse | 5'-GCTTGGCGAAGAAATCGGAGA-3' |
| p19 | Forward | 5'-AGTCCAGTCCATGACGCAG-3' |
|  | Reverse | 5'-ATCAGGCACGTTGACATCAGC-3' |
| p27 | Forward | 5'-TAATTGGGGCTCCGGCTAACT-3' |
|  | Reverse | 5'-TGCAGGTCGCTTCCTTATTCC -3' |
| p18 | Forward | 5'- CGAAACCAGTTCGGTCTTTCAA-3' |
|  | Reverse | 5'-CAGCGCAGTCCTTCCAAAT-3' |
| RPL15 | Forward | 5'-CCCACCCGGCCTGATAAAG-3' |
|  | Reverse | 5'-CACGGCGAACACGAATCCT-3' |
| RPL36 | Forward | 5'-ATGGCCCTACGCTACCCTATG-3' |
|  | Reverse | 5'-CCTCCCGAATCATGTCCCG-3' |
| RPL13 | Forward | 5'-TCAAAGCCTTCGCTAGTCTCC-3' |
|  | Reverse | 5'-GGCTCTTTTTGCCCGTATGC-3' |
| RPL31 | Forward | 5'-CTCGGGCACTCAAAGAGATTC-3' |
|  | Reverse | 5'-CGGATTCGGTATGGCACATTC-3' |
| RPL22 | Forward | 5'-AAAGTGAACGGAAAAGCTGGG-3' |
|  | Reverse | 5'-TCACGGTGATCTTGCTCTTGC-3' |
| RPL3 | Forward | 5'-CTACCATCACCGCACTGAGAT-3' |
|  | Reverse | 5'-GGTCACTTCACCATAGTGGACA-3' |
| RPS16 | Forward | 5'-TCGGACGCAAGAAGACAGC-3' |
|  | Reverse | 5'-AGCAGCTTGTACTGTAGCGTG-3' |
| RPS2 | Forward | 5'-CGTCGGTCTGGGTGTTAAGTG-3' |
|  | Reverse | 5'-GGCTTGCCGATCTTGTTCC-3' |
| RPS5 | Forward | 5'-ATGACCGAGTGGGAGACAG-3' |
|  | Reverse | 5'-GCTTTGCGGAAGCGTTTGG-3' |
| RPS19 | Forward | 5'-AAGCTGAAAGTCCCCGAATGG-3' |
|  | Reverse | 5'-AGTTCTCATCGTAGGGAGCAAG-3' |
| RPS27 | Forward | 5'-ATGCCTCTCGCAAAGGATCTC-3' |
|  | Reverse | 5'-TGAAGTAGGAATTGGGGCTCT-3' |

**Supplemental table 1.** The qRT-PCR primer sequence.

| Gene | Sequence | |
| --- | --- | --- |
| p21 | Forward | 5'-AGGTATTTAGAGGAGGTGAGA-3' |
|  | Reverse | 5'-ACCAAAAATTCCTATACTTAT-3' |
| RPL15 | Forward | 5'-GGTTTTTATGTTAGTAAATTTGGGA-3' |
|  | Reverse | 5'-AAAACTACACTAAAAAATCCCC-3' |
| TP53 | Forward | 5'-TTTGGGAGTAGGTAGAAGAT-3' |
|  | Reverse | 5'-AAAAACCTTCTAACCTTTCAC-3' |

**Supplemental table 2.** The bisulfite sequencing primers sequence for p21, RPL15 and TP53.

| Gene | Sequence |
| --- | --- |
| cas9-sgRNA-F | 5'-CACCGCAATTCCGACTCGACCTATG-3' |
| cas9-sgRNA-R | 5'-AAACCATAGGTCGAGTCGGAATTGC-3' |
| dcas9-dsgRNA-F | 5'-CACCGGTTTCCGCGCGAAAAGCCG-3' |
| dcas9-dsgRNA-R | 5'-AAACCGGCTTTTCGCGCGGAAACC-3' |

**Supplemental table 3.** The sgRNA and dsgRNA sequence targeted DNMT1.
